# Comparison of Effects of p53 Null and Gain-of-Function Mutations on Salivary Tumors in MMTV-*Hras* Transgenic Mice

**DOI:** 10.1371/journal.pone.0118029

**Published:** 2015-02-19

**Authors:** Dadi Jiang, Catherine I. Dumur, H. Davis Massey, Viswanathan Ramakrishnan, Mark A. Subler, Jolene J. Windle

**Affiliations:** 1 Department of Human and Molecular Genetics, Virginia Commonwealth University, Richmond, Virginia, United States of America; 2 Department of Pathology, Virginia Commonwealth University, Richmond, Virginia, United States of America; 3 Massey Cancer Center, Virginia Commonwealth University, Richmond, Virginia, United States of America; 4 Department of Public Health Sciences, Medical University of South Carolina, Charleston, South Carolina, United States of America; National Cancer Center Research Institute, JAPAN

## Abstract

*p53* is an important tumor suppressor gene which is mutated in ~50% of all human cancers. Some of these mutants appear to have acquired novel functions beyond merely losing wild-type functions. To investigate these gain-of-function effects *in vivo*, we generated mice of three different genotypes: MMTV-*Hras/p53^+/+^*, MMTV-*Hras/p53^-/-^*, and MMTV-*Hras/p53^R172H/R172H^*. Salivary tumors from these mice were characterized with regard to age of tumor onset, tumor growth rates, cell cycle distribution, apoptotic levels, tumor histopathology, as well as response to doxorubicin treatment. Microarray analysis was also performed to profile gene expression. The MMTV-*Hras/p53^-/-^* and MMTV-*Hras*/*p53^R172H/R172H^* mice displayed similar properties with regard to age of tumor onset, tumor growth rates, tumor histopathology, and response to doxorubicin, while both groups were clearly distinct from the MMTV-*Hras/p53^+/+^* mice by these measurements. In addition, the gene expression profiles of the MMTV-*Hras/p53^-/-^* and MMTV-*Hras*/*p53^R172H/R172H^* tumors were tightly clustered, and clearly distinct from the profiles of the MMTV-*Hras/p53^+/+^* tumors. Only a small group of genes showing differential expression between the MMTV-*Hras*/*p53^-/-^* and MMTV-*Hras*/*p53^R172H/R172H^* tumors, that did not appear to be regulated by wild-type p53, were identified. Taken together, these results indicate that in this MMTV-*Hras*-driven salivary tumor model, the major effect of the p53 R172H mutant is due to the loss of wild-type p53 function, with little or no gain-of-function effect on tumorigenesis, which may be explained by the tissue- and tumor type-specific properties of this gain-of-function mutant of p53.

## Introduction

p53 is widely regarded as one of the most important tumor suppressor genes, as it is mutated in over 50% of all human cancers, and the p53 pathway is found to be inactivated in most if not all tumors [1]. p53 knock-out mice are largely normal at birth, indicating a non-essential role for p53 during embryonic development. However, the mice are highly tumor-prone, with the majority developing lymphomas and sarcomas by 6 months of age, underscoring p53’s essential tumor suppressor function [2–5]. The p53 protein executes its tumor-suppressive functions primarily through its role as a sequence-specific transcription factor [6]. Upon stabilization and activation by oncogenic signals or other types of cellular stresses, including DNA damage, hypoxia, nutrient deprivation and reactive oxygen species (ROS), p53 transactivates or transrepresses a panel of downstream effector genes which are involved in mediating multiple cellular responses, including transient G_1_ cell cycle arrest, DNA repair, cellular senescence characterized by a permanent cell cycle arrest, and apoptosis [2,7].

Most p53 mutations arise somatically during tumor development and progression. However, p53 mutations can also be transmitted in the germ-line and give rise to cancer predisposition conditions called Li-Fraumeni Syndrome (LFS) and Li-Fraumeni-like Syndrome (LFL) [8–10]. Unlike changes in other classical tumor suppressor genes during tumorigenesis, which frequently involve frame-shift or nonsense mutations, nearly three-quarters of both somatic and germ-line p53 mutations are missense mutations, according to the latest release (R17) of the IARC p53 mutation database [1] (also see http://www-p53.iarc.fr/). The vast majority of these missense mutations are clustered in the central DNA-binding domain of the p53 protein, either affecting residues directly contacting DNA (DNA contact mutations), or those important for maintaining the conformation of the DNA-binding domain (conformational or structural mutations). Both classes of mutations disrupt the sequence-specific DNA-binding function of the p53 protein, thus preventing p53 from acting as a transcription factor [11,12]. In addition to loss of the wild-type functions, some p53 mutants also demonstrate dominant-negative effects over the remaining wild-type allele, preventing the wild-type p53 protein from inhibiting cellular transformation [13,14]. Further, a subset of p53 missense mutants have been shown to possess a variety of novel gain-of-function properties. When expressed in a p53-null background, these p53 mutants confer accelerated cell growth *in vitro* [15–21], increased tumorigenicity in mouse xenograft models [16,20,22–24], anti-apoptotic effects and chemoresistance [25–30], exacerbated genomic instability [25,31–33], enhanced somatic cell programming [34], disruption of tissue architecture [35], and increased migration, invasion and metastasis [16,36,37]. Although the mechanism(s) contributing to these gain-of-function effects are still under investigation, several models have been proposed. Firstly, a subset of tumor-derived p53 mutants physically interact with a host of cellular proteins such as p63/73, MRE11, PML and Pin1 [38]. Interaction between mutant p53 and the p53 family members p63/73 leads to altered activities of these sequence-specific transcription factors and contributes to promotion of chemoresistance, migration, invasion and metastasis [36,37]. Alternatively, mutant p53 may also transcriptionally regulate a novel set of genes, many of which are involved in increasing cell proliferation, inhibiting apoptosis, promoting chemoresistance, and regulating metabolism as well as cell-cell/cell-ECM signaling pathways [13,38–40]. The altered target affinity in transcriptional regulation by mutant p53 is postulated to be mediated through interaction with other sequence-specific transcription factors, thus inducing or repressing their target gene expression.

Although the majority of studies characterizing the gain-of-function properties of mutant p53 have been conducted using cell culture systems, a variety of genetically engineered mouse tumor models have also been developed for examining the effects of mutant p53 *in vivo*. For example, when p53 R172H, which is equivalent to the hot-spot human p53 R175H mutant (a conformational mutation), was expressed in the epidermis [41] or mammary glands [42] of transgenic mice, accelerated carcinogen-induced skin or mammary tumorigenesis was observed in the corresponding mouse models. These mutant p53-expressing tumors also demonstrated features of advanced malignancy when compared to wild-type p53-expressing counterparts. Knock-in mouse models of p53 R172H and R270H (a hot-spot DNA contact mutant, equivalent to human R273H) have also been generated by two different groups, allowing for a comparison of the effects of null versus gain-of-function mutants on the tumor phenotype of these mice [45,46]. Although survival time was not significantly different between mice harboring the mutant p53 alleles and p53-deficient mice, mutant p53 knock-in mice displayed an altered tumor spectrum, with a greater number of mice developing carcinomas, and the tumors that arose metastasized with a much higher frequency [43,44].

Although the mutant p53 knock-in mice allowed for the comparison of tumorigenesis and tumor properties in mice carrying p53 null and p53 gain-of-function alleles, direct comparisons within a particular tumor type were complicated by the fact that mice in these models develop multiple tumor types. We therefore sought to create animal models that would allow for a head-to-head comparison of the effects of p53 null and gain-of-function mutations within the context of a single tumor type. Our lab previously utilized the MMTV-v-Ha-*ras* (MMTV-*Hras*) transgenic mouse mammary/salivary tumor model [45] crossed with p53 null mice [3] to study the influence of p53 loss on tumorigenesis and tumor properties. While the MMTV-*Hras/p53* ^*+/+*^ mice develop both mammary and salivary tumors, salivary tumorigenesis was greatly accelerated in the MMTV-*Hras/p53* ^*-/-*^ mice. The MMTV-*Hras/p53* ^*-/-*^ salivary tumors had higher histopathological grades, growth rates, S-phase fractions, and genomic instability than the MMTV-*Hras/p53* ^*+/+*^ tumors [46]. In addition, the MMTV-*Hras/p53* ^*+/+*^ tumors responded better to doxorubicin treatment than the MMTV-*Hras/p53* ^*-/-*^ tumors, due to a decrease in S phase fraction and an increase in G_1_ phase fraction, effects which were absent in the treated MMTV-*Hras/p53* ^*-/-*^ tumors [47]. No significant difference in apoptotic levels was identified in this tumor model, regardless of the *p53* status and treatment, likely due to the anti-apoptotic effects of the activated Ras signaling pathways driving tumorigenesis in this model.

In the current study, we have included p53 R172H as the third p53 status in this tumor model by crossing the *MMTV-Hras* mice to p53 R172H knock-in mice [46], and have performed head-to-head comparisons of the effects of the three classes of p53 status on salivary tumorigenesis, tumor properties, and tumor response to doxorubicin. While the MMTV-*Hras/p53* ^*+/+*^ tumors differed significantly from both the MMTV-*Hras/p53* ^*-/-*^ and MMTV-*Hras/p53* ^*R172H/R172H*^ tumors in these measurements, p53 null and R172H tumors exhibited very similar properties. We also performed gene expression profiling on salivary tumors of the three genotypes and identified the differentially regulated genes among the three classes. Again, the p53^+/+^ tumors clustered separately from the other two classes, but there were relatively few gene expression differences between the p53 null and R172H tumors. These results indicate that, within the context of activated Ha-*ras* expression in the mouse salivary gland, the primary effect of p53 R172H mutation is the loss of wild-type p53 function, with little discernable gain-of-function effect on tumorigenesis.

## Materials and Methods

### Ethics Statement

All animal studies and care were performed under the guidelines of the Virginia Commonwealth University (VCU) Institutional Animal Care and Use Committee (IACUC), in accordance with the principles and procedures outlined in the National Research Council “Guide for the Care and Use of Laboratory Animals” under Assurance Number A3281-01. Specific approval for these studies was granted by the VCU IACUC under protocol #AM10313, entitled “Molecular Genetics of Tumorigenesis in Transgenic Mice”. Pain and distress resulting from tumor development was minimized by euthanizing any mouse with a palpable tumor of 2500mm^3^ or displaying indications of morbidity.

### Generation of MMTV-*Hras*, MMTV-*Hras/p53* ^*-/-*^ and MMTV-*Hras/p53* ^*R172H/R172H*^ mice

MMTV-*Hras* and MMTV-*Hras/p53* ^*-/-*^ mice were generated as described in [46], and have been maintained on a Balb/c x C57BL/6 (CB6) genetic background for >30 generations (including multiple generations of breeding of colony mice to purchased CB6F1 mice.) *p53* ^*R172H/+*^ mice were generously provided by Dr. Gigi Lozano (The University of Texas MD Anderson Cancer Center) [44]. To generate MMTV-*Hras/p53* ^*R172H/R172H*^ mice, *p53* ^*R172H/+*^ mice on a C57BL/6 background were initially mated to MMTV-*Hras/p53* ^*+/-*^ mice on a CB6 background. The colony was subsequently maintained on the C57BL/6 x BALB/c mixed background by interbreeding of mice from this colony. Mice were genotyped by PCR for determination of *ras* transgene and p53 status [44,46].

### Identification of salivary tumor onset and measurement of tumor weight

Mice were monitored visually or by palpation 2–3 times weekly for the presence of tumors arising from the salivary glands (parotid, submandibular, and sublingual glands). Once a tumor was detected, tumor size was measured (3 times per week initially and daily as the tumor became larger) by caliper measurement of the width and length of the tumor. Tumor weight was estimated using the formula: tumor weight (mg) = (L x W^2^)/2, where L is the length and W is the width of the tumor in mm. Tumor-bearing mice were sacrificed when the weight of the tumor approached 2500 mg (or smaller for some studies) and the tumor was dissected out. The tumor mass was then cut into multiple sections, one of which was placed in 10% formalin and the rest into 1.2 ml cryovials which were then flash frozen in liquid nitrogen and stored at -80°.

### Analysis of age of tumor onset and tumor growth rates

Age of tumor onset data were recorded for all male MMTV-*Hras/p53* ^*+/+*^, MMTV-*Hras/p53* ^*-/-*^, and MMTV-*Hras/p53* ^*R172H/R172H*^ mice. The survival function was used to calculate the salivary tumor-free survival for each group of mice using GraphPad Prism 4. Mice that died or were sacrificed without developing a salivary tumor were censored in the survival analysis. Only tumors with initial palpable size <500 mg were used to calculate mean tumor weights for plotting tumor growth curves.

### Immunoblotting and immunohistochemistry

For immunoblotting, tumor protein was extracted from frozen tissue using PIERCE RIPA buffer, supplemented with additional 1X protease inhibitor cocktail containing 50mM NaF, 1mM Na_3_VO_4_, 17.5mM β-glycerophosphate, and 1mM PMSF (Calbiochem). For immunohistochemistry, tumor tissues fixed in 10% phosphate-buffered formalin were transferred to 70% ethanol after 48 hours to preserve antigens. Fixed tumor tissues were paraffin embedded and cut into 5μm sections for staining. Primary antibodies and dilution used for immunoblotting include: p53 (CM5, Novocastra) at 1:1000; p19ARF (ab80, Abcam) at 1:1000; p16 (M-156, Santa Cruz Biotech) at 1:1000; p15 (K-18, Santa Cruz Biotech) at 1:1000 and Actin (C-11, Santa Cruz Biotech) at 1:1000. HRP-conjugated Secondary antibodies (Jackson ImmunoResearch Laboratories) were used at 1:2000 dilution. An enhanced chemiluminescence (ECL) assay (PerkinElmer) was used and documented either on Kodak X-Omat Blue XB-1 film or by an AlphaEase digital imaging system (Alpha Innotech). Primary antibodies and dilution used for immunohistochemistry include: Ki-67 (Clone SP6, Lab Vision) at 1:200; cleaved caspase 3 (Biocare Medical) at 1:100; p53 (CM5, Novocastra) at 1:100.

### Characterization of tumor response to doxorubicin

A subset of salivary tumor-bearing mice in each genotypic group were treated with doxorubicin. When the weight of the tumor reached 500–700 mg, the mouse was subjected to a 9-day treatment schedule at a dose of 2 mg/kg doxorubicin/day, injected IP. During the treatment period, tumor dimensions were measured daily and tumor weight calculated the same way as in the tumor growth analysis. Only tumors with initial palpable size <800 mg were used to calculate mean tumor weights for plotting tumor growth curves.

### Gene expression profiling by microarray analysis

For RNA extraction, 20–40 10 μm frozen sections from each candidate tumor were collected. RNA was extracted using TRIZOL reagent (Invitrogen Life Technologies), according to the manufacture’s protocols. Total RNA isolated from the tumor sections was subjected to a cleanup step using the RNeasy Mini Kit (Qiagen). Microarray analysis was performed using the Affymetrix GeneChip platform and the Mouse 430A 2.0 array according to the Affymetrix standard protocol. Multiple levels of data analysis were performed in the BRB-ArrayTools software, version 3.5.0 (Richard Simon & Amy Peng Lam, Biometric Research Branch, Division of Cancer Treatment and Diagnosis, NCI). The probe-level microarray data (in .CEL files) were collated in BRB-ArrayTools using the RMA (Robust Multi-chip Average) method. Microarray data used in this study are deposited in the Gene Expression Ominibus (GEO) database with accession # GSE59452.

### Validation of microarray data by quantitative PCR

3 μg of total RNA from each tumor sample was loaded in each RT reaction, using the SuperScript III First-strand Synthesis system (Invitrogen Inc.) according to the manufacture’s protocol, and oligo(dT) was selected to prime the RT reactions. Primers for the real-time PCR assays were designed using the Primer Express Version 3.0 software (Applied Biosystems) using the standard melting temperature (60°C) and at least one of the two primers for each gene was selected to span an exon-intron boundary, to avoid amplifying genomic DNA contaminants. The real-time PCR assay was performed on the Applied Biosystems 7500 Real-time PCR System (Applied Biosystems). The Relative Standard Curves method was used as the study design for the quantitative PCR assay (Real-Time PCR Systems Chemistry Guide, Applied Biosystems). β-actin was used as the internal control. The raw data were analyzed in the Sequence Detection Software Version 1.3.1 (Applied Biosystems) according to the manufacture’s protocol. Sequences of the primers used in the quantitative PCR assays are listed in S7 Table.

## Results

### Age of tumor onset for mice of each p53 genotype

To determine the potential differential effects of p53 loss-of-function and gain-of-function mutations on tumor phenotype within the context of a single tissue of origin (salivary tumors arising in MMTV-*Hras* male transgenic mice), we generated cohorts of mice of the following genotypes: MMTV-*Hras/p53* ^*+/+*^, MMTV-*Hras/p53* ^*-/-*^, and MMTV-*Hras/p53* ^*R172H/R172H*^. Although female MMTV-*Hras/p53* ^*+/+*^ mice develop primarily mammary tumors, male mice of this genotype develop primarily salivary tumors (at a later average age of tumor onset). However, we have previously shown that when bred to p53 knock-out mice, both males and females of the MMTV-*Hras/p53* ^*-/-*^ genotype develop primarily salivary tumors due to an acceleration of salivary but not mammary tumorigenesis in this model [46]. We observed a similar prevalence of salivary tumorigenesis in the MMTV-*Hras/p53* ^*R172H/R172H*^ mice. Therefore, in order to compare tumors of a single cell type of origin arising in mice of a single gender, we restricted our analysis to salivary tumorigenesis in male mice of each of the three genotypes. (A small number of female mice were also used for some of the analyses, and no recognizable difference in the salivary tumor properties characterized between the two genders was observed.)

**Fig 1:**
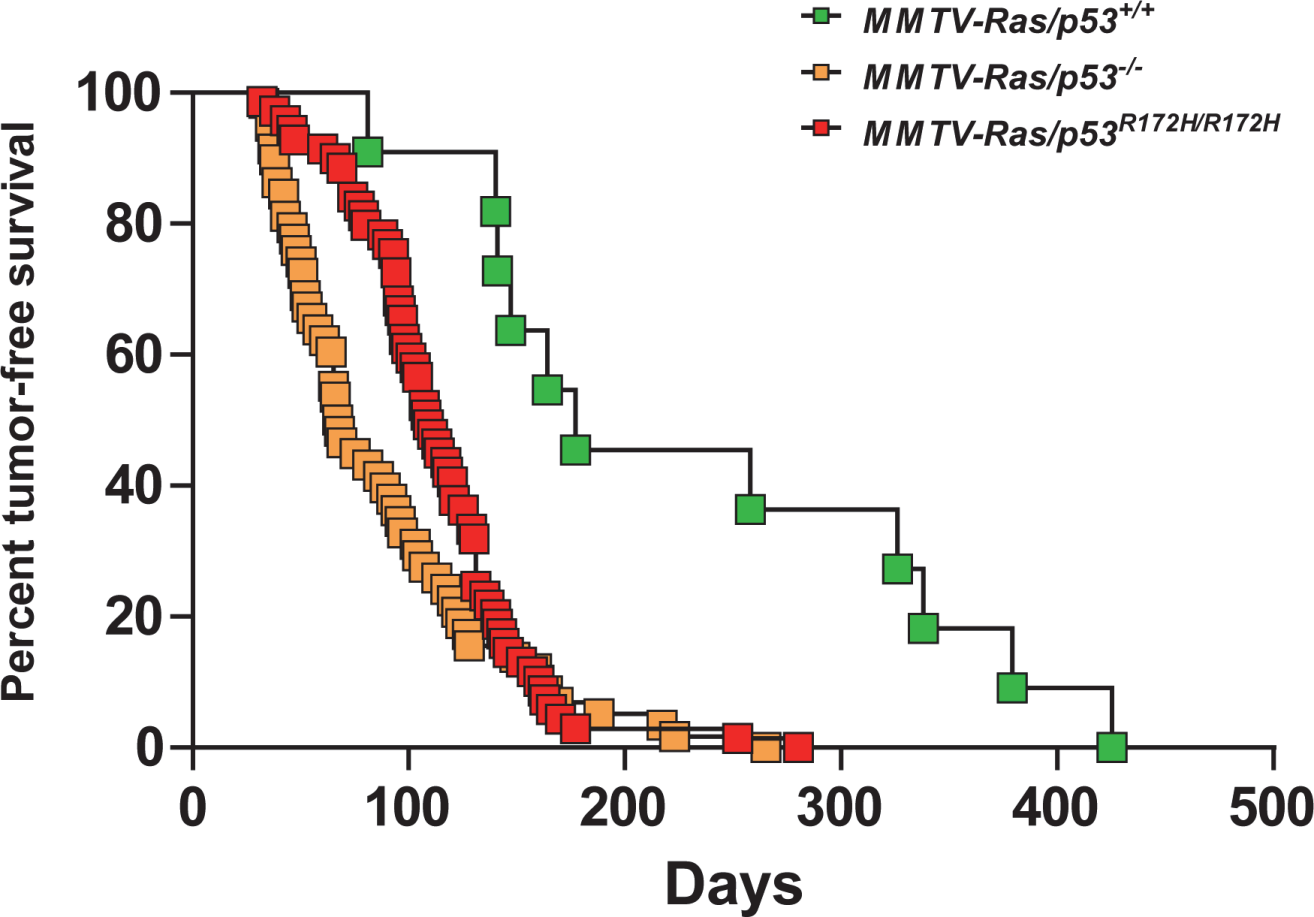
Kaplan-Meier analysis of salivary tumor-free survival. The proportion of tumor-free mice as a function of time for MMTV-*Hras/p53* ^*+/+*^ (green), MMTV-*Hras/p53* ^*-/-*^ (yellow), and MMTV-*Hras/p53* ^*R172H/R172H*^ (red) male mice are shown, based upon the age at which a palpable salivary tumor was first detected.

S1 Table summarizes the mice used for the age of tumor onset analysis. Significant fractions of the *p53* ^*-/-*^ and *p53* ^*R172H/R172H*^ mice developed symptoms indicating the presence of lymphomas and/or sarcomas prior to the development of salivary tumors, consistent with previous reports of the prevalence of these tumor types in *p53* ^*-/-*^ and *p53* ^*R172H/R172H*^ mice lacking the MMTV-*Hras* transgene [2–5,43,44], which necessitated sacrifice of the mice. Because the tumor spectrum in both *p53* ^*-/-*^ and *p53* ^*R172H/R172H*^ mice has already been well-characterized [3,4,43,44,48], we did not analyze tumor types in animals lacking salivary tumors. In addition, 38 MMTV-*Hras/p53* ^*+/+*^ mice (of a total of 114) without salivary tumors were sacrificed due to development of Harderian gland tumors, consistent with previous reports [45]. Animals developing lymphomas, sarcomas or Harderian gland tumors were censored from the dataset used for Kaplan-Meier analysis. With a similar total starting number of mice in each genotypic group, the mean ages of salivary tumor onset in the MMTV-*Hras/p53* ^*-/-*^ and MMTV-*Hras/p53* ^*R172H/R172H*^ mice were much earlier than in the MMTV-*Hras/p53* ^*+/+*^ mice and the overall salivary tumor incidence was much higher than in the MMTV-*Hras/p53* ^*+/+*^ mice. As shown in Fig. 1 and S1 Table, the median time to salivary tumor onset was 177 days for the MMTV- *Hras/p53* ^*+/+*^ mice, 68 days for the MMTV-*Hras/p53* ^*-/-*^ mice and 109 days for the MMTV-*Hras/p53* ^*R172H/R172H*^ mice. We observed a statistically significant difference between the MMTV-*Hras/p53* ^*-/-*^ and MMTV-*Hras/p53* ^*R172H/R172H*^ mice in age of tumor onset (*p* = 0.0261, Log-rank test), with a slightly delayed tumor onset in the MMTV-*Hras/p53* ^*R172H/R172H*^ mice. However, both the MMTV-*Hras/p53* ^*-/-*^ and MMTV-*Hras/p53* ^*R172H/R172H*^ mice developed salivary tumors much more rapidly than the MMTV-*Hras/p53* ^*+/+*^ mice (*p*<0.0001, Log-rank test).

To exclude the possibility that tumors from the MMTV-*Hras/p53* ^*+/+*^ mice had acquired somatic mutations in the *p53* gene which might confound our subsequent analyses, exons 5–9 of the *p53* locus of 4 MMTV-*Hras/p53* ^*+/+*^ salivary tumors (all of which were used for the subsequent microarray analysis) were sequenced, and no *p53* mutations were detected (data not shown).

### Growth rates for tumors of each p53 genotype

**Fig 2:**
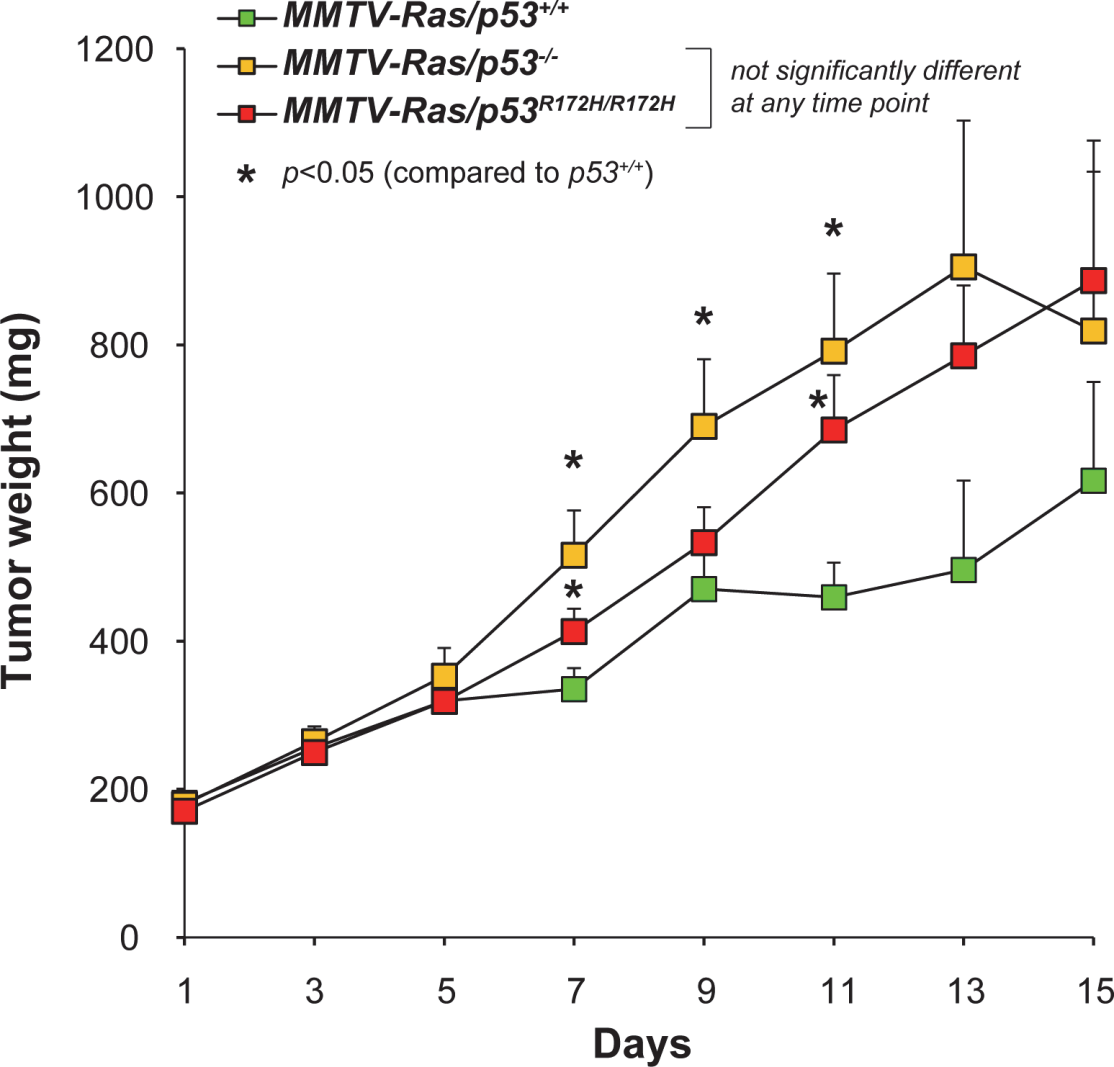
Tumor growth rates. Tumor growth rates over time (days) measured as calculated weight (mg) for each of the three groups of tumors were plotted. Each data point represents the mean ± SEM.

We have previously shown that salivary tumors arising in MMTV-*Hras/p53* ^*-/-*^ mice have growth rates that are nearly double those of salivary tumors arising in MMTV-*Hras/p53* ^*+/+*^ mice [46]. To determine whether a p53 gain-of-function mutation further accelerates the tumor growth rate, the growth of multiple independent salivary tumors arising in mice of each of the three different genotypes (MMTV-*Hras/p53* ^*+/+*^, MMTV-*Hras/p53* ^*-/-*^, and MMTV-*Hras/p53* ^*R172H/R172H*^) was compared (Fig. 2; S1 Fig.). Tumor growth data from 26 MMTV-*Hras/p53* ^*+/+*^ tumors (16 from males and 10 from females), 34 MMTV-*Hras/p53* ^*-/-*^ tumors (21 from males and 13 from females), and 39 MMTV-*Hras/p53* ^*R172H/R172H*^ tumors (35 from males and 4 from females) was obtained. The mean tumor growth curves for each genotype are shown in Fig. 2, and the individual tumor growth curves are shown in S1 Fig. The growth of most of the salivary tumors roughly followed an exponential growth pattern, which indicates the existence of constant doubling times of these tumors. The MMTV-*Hras/p53* ^*+/+*^ tumors grew significantly slower than the MMTV-*Hras/p53* ^*-/-*^ and MMTV-*Hras/p53* ^*R172H/R172H*^ tumors, as was previously reported for the comparison of MMTV-*Hras/p53* ^*+/+*^ and *Hras/p53* ^*-/-*^ tumors [46]. However, there was no significant difference between the growth rates of the *Hras/p53* ^*-/-*^ and MMTV-*Hras/p53* ^*R172H/R172H*^ tumors.

### p53 protein levels in tumors of each genotype

**Fig 3:**
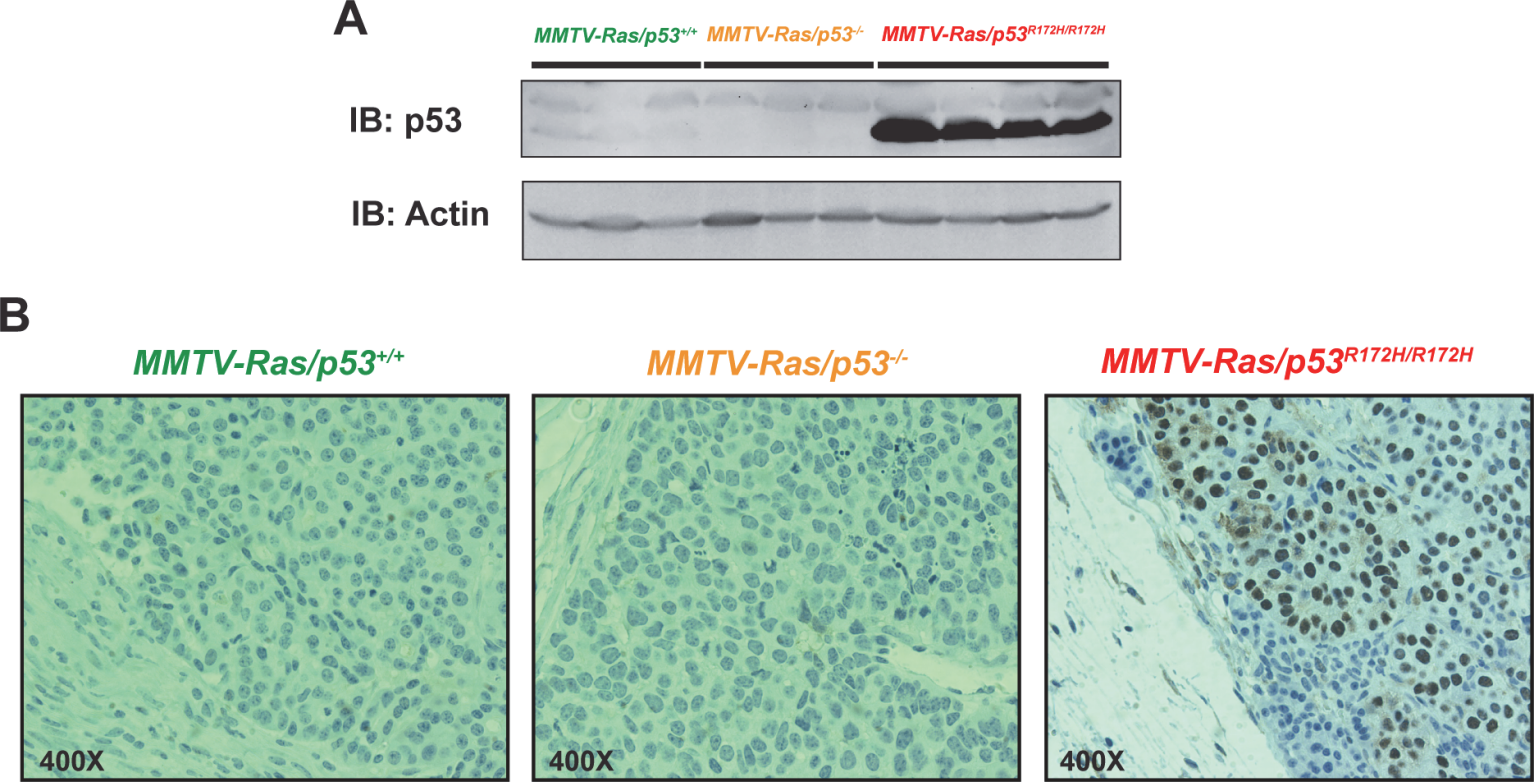
p53 protein levels in tumors of each genotype. (A) Western blot analysis of p53 using an antibody that detects both wild-type and mutant p53 (CM5). From left to right: 3 MMTV-*Hras/p53* ^*+/+*^, 3 MMTV-*Hras/p53* ^*-/-*^, and 4 MMTV-*Hras/p53* ^*R172H/R172H*^ tumors. (B) Immunohistochemical staining with the CM5 anti-p53 antibody in a representative tumor of each of the three genotypes.

To confirm that mutant p53 was expressed and stabilized in the MMTV-*Hras/p53* ^*R172H/R172H*^ tumors, and to characterize the expression patterns of mutant p53 protein in the tumor tissues, western blotting and immunohistochemical analysis were performed on tumors of each genotype, using an antibody that detects both wild-type and mutant p53. Western blot analysis showed that mutant p53 was present in very high levels in all of the MMTV-*Hras/p53* ^*R172H/R172H*^ tumors tested, whereas p53 was barely detectable in the MMTV-*Hras/p53* ^*+/+*^ tumors and completely absent in the MMTV-*Hras/p53* ^*-/-*^ tumors (Fig. 3A). Immunohistochemical staining with the same antibody on the tumor sections showed that while p53 was undetectable in both MMTV-*Hras/p53* ^*+/+*^ and MMTV-*Hras/p53* ^*-/-*^ tumors, the MMTV-*Hras/p53* ^*R172H/R172H*^ tumors displayed a relatively uniform, high level of p53 in the nucleus of tumor cells, but not in the adjacent stromal cells (Fig. 3B).

### Tumor histology

**Fig 4:**
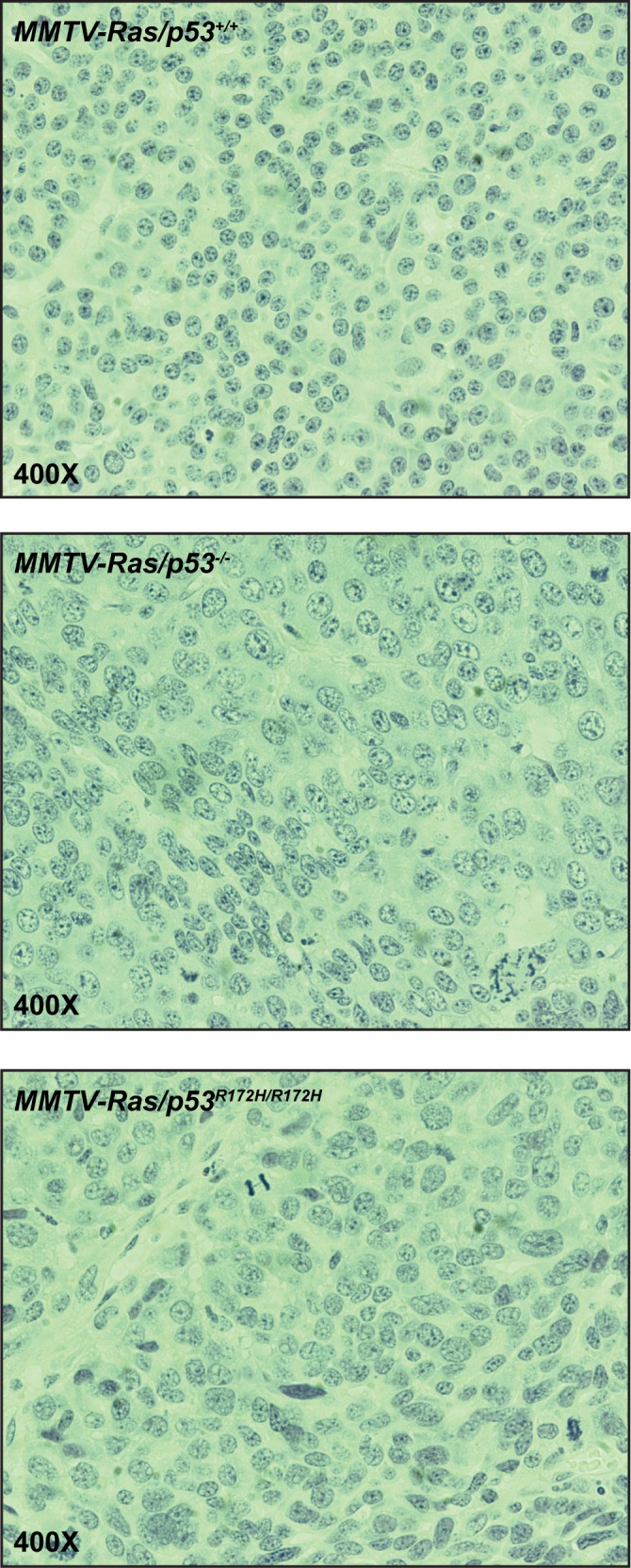
H&E staining of representative tumor of the three genotypes. Top: MMTV-*Hras/p53* ^*+/+*^; middle: MMTV-*Hras/p53* ^*-/-*^; bottom: MMTV-*Hras/p53* ^*R172H/R172H*^.

We have previously shown that in this MMTV-*Hras* transgenic mouse tumor model, absence of p53 resulted in tumors with higher histologic grades [46]. To determine whether p53 loss-of-function and gain-of-function mutations differentially affected tumor histopathology, formalin-fixed tumor samples from the three genotypic groups were embedded in paraffin blocks, and sections were stained with hematoxylin and eosin and subjected to microscopic evaluation. A set of morphological parameters were evaluated using a 3-point grading system. We assessed the nuclear/cytoplasmic ratio, the degree of nuclear pleomorphism, and overall tumor architecture. We also took in account the presence of a spindle cell morphology, and whether “giant cells”, which display remarkably enlarged cell and nucleus areas, were present. Mitotic activity, percentage of tumors with apoptotic cells, and degree of necrosis were also estimated. The results are summarized in S2 Fig. In general, tumors of each of the three genotypes were well-localized and encapsulated with no signs of metastasis, and were composed of nests and cords of poorly differentiated carcinoma cells. The MMTV-*Hras/p53* ^*+/+*^ tumor cells were relatively uniform in size and shape, while both the MMTV-*Hras/p53* ^*-/-*^ and MMTV-*Hras/p53* ^*R172H/R172H*^ tumor cells displayed more variability in cell and nuclear size and shape and less evenly distributed chromatin in the nucleus (Fig. 4). When nuclear/cytoplasmic ratio, level of pleomorphism, and cellular architecture were measured using a 3-point grading system, the MMTV-*Hras/p53* ^*+/+*^ tumors were assigned a lower grade on average than the MMTV-*Hras/p53* ^*-/-*^ and MMTV-*Hras/p53* ^*R172H/R172H*^ tumors, although the differences were not statistically significant (S2 Fig. A). The MMTV-*Hras/p53* ^*+/+*^ tumors contained fewer cells with mitotic figures than the MMTV-*Hras/p53* ^*-/-*^ and MMTV-*Hras/p53* ^*R172H/R172H*^ tumors, while the latter two groups were indistinguishable (S2 Fig. E). It is also noticeable that there were high levels of variation in all of the four measurements, indicating the existence of significant heterogeneity in the histopathology of these tumors. Proportions of tumors displaying features of spindling (S2 Fig. B) and apoptosis (S2 Fig. D), as well as those containing “giant cells” with greatly enlarged cell and nuclear size are also summarized (S2 Fig. C). Similarly higher fractions of the MMTV-*Hras/p53* ^*-/-*^ and MMTV-*Hras/p53* ^*R172H/R172H*^ tumors demonstrated features of spindling than the MMTV-*Hras/p53* ^*+/+*^ tumors. Interestingly, the “giant cells” represented a slightly higher fraction of the tumor cells in the MMTV-*Hras/p53* ^*R172H/R172H*^ tumors than in the other two groups of tumors. There was no significant difference in the fractions of tumors demonstrating apoptosis among the three genotypic groups. As the levels of apoptosis in individual tumors were not taken into account in this analysis, the result has limited value as to measure the representative apoptotic levels. The levels of necrosis were also estimated. Compared to the MMTV-*Hras/p53* ^*+/+*^ and MMTV-*Hras/p53* ^*-/-*^ tumors, the MMTV-Hras/*p53* ^*R172H/R172H*^ tumors displayed higher levels of necrosis (S2 Fig. F).

### Cell cycle and apoptosis studies

We previously showed that MMTV-*Hras/p53* ^*-/-*^ tumors had a significantly higher fraction of cells in the S and G_2_/M phases of the cell cycle, with a corresponding decrease in the fraction of cells in G_1_, compared to MMTV-*Hras/p53* ^*+/+*^ tumors [46]. To compare the effect of p53 loss-of-function and gain-of-function mutations on tumor cell proliferation, tumors of each genotype were immunohistochemically stained with an antibody for the proliferation marker Ki-67. However, there was extensive heterogeneity in the Ki-67 staining across the entire set of tumor samples. Frequently, tumors from the same genotypic group (S3 Fig. A), or even different regions of the same tumor (S3 Fig. B), displayed highly variable fractions of Ki-67-positive cells. The staining ranged from a complete lack of staining to staining of almost every cell. This variation hampered accurate assessments of the representative fraction of proliferating cells of each genotypic group. However, there were no grossly recognizable differences in the overall staining patterns of the three groups of tumors.

Previous studies also demonstrated that apoptosis levels were constitutively low in the tumors with the MMTV-*Hras* transgene regardless of whether they were *p53* ^*+/+*^ or *p53* ^*-/-*^, likely due to the anti-apoptotic effects of the pathways downstream of activated Ras [46]. To investigate whether the same scenario holds true for tumors bearing the *p53* ^*R172H*^ mutation, we examined the levels of spontaneous apoptosis in the three groups of tumors by staining the tumors immunohistochemically with an antibody specific for cleaved caspase 3 (Cc3), which is the activated version of a key apoptosis mediator. As for the Ki-67 staining, there was also significant variation in the staining patterns for Cc3, either among different tumors, or among different regions of the same tumor. Cc3 immunostaining revealed low indices of apoptosis in all three genotypic groups of tumors (data not shown).

### Tumor response to doxorubicin

**Fig 5:**
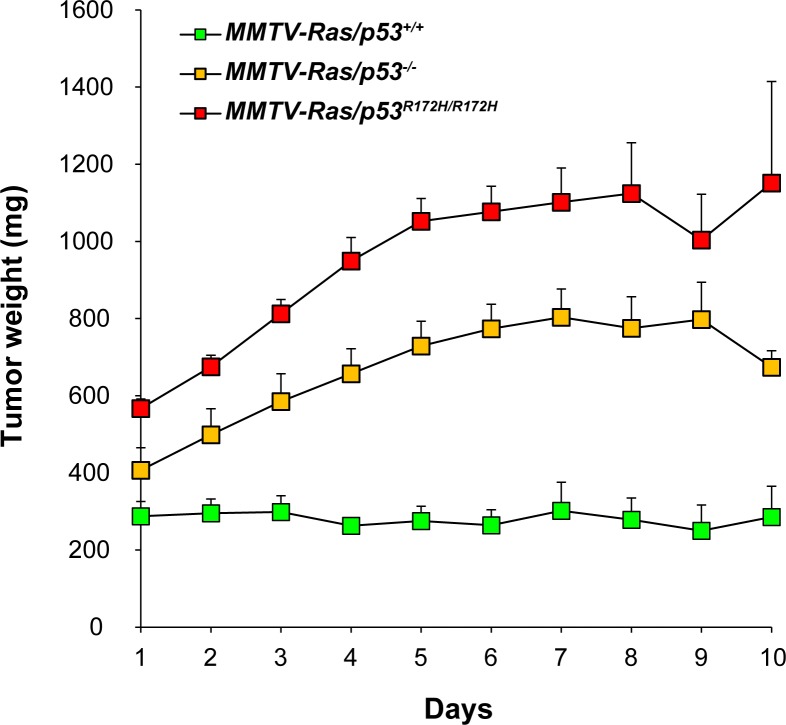
Tumor growth responses to doxorubicin. Tumor-bearing mice were treated for nine consecutive days with doxorubicin (2 mg/kg), and tumor growth was monitored daily by caliper measurements. Tumor growth rates over time (days) measured as calculated weight (mg) for each of the three groups of tumors were plotted. Each data point represents the mean ± SEM.

It is well established that p53 mutation confers resistance of tumor cells to a wide range of chemotherapeutic agents [38], and several studies have demonstrated increased chemoresistance in tumors expressing gain-of-function p53 mutants compared to those lacking p53 altogether [17,20,21,25,29]. We have previously shown that tumors arising in MMTV-*Hras/p53* ^*-/-*^ mice are significantly impaired in their response to doxorubicin compared to those arising in MMTV-*Hras/p53* ^*+/+*^ mice [47]. To determine whether the p53 R172H gain-of-function mutation promoted further resistance to doxorubicin, a subset of salivary tumor-bearing mice from each of the three genotypic groups was treated with doxorubicin for 9 consecutive days, and the effects on tumor growth were measured (Fig. 5; S4 Fig.). As we previously reported, the growth of MMTV-*Hras/p53* ^*+/+*^ tumors plateaued following the first day of treatment, with some of the tumors actually regressing during the 9-day treatment period. In contrast, both the MMTV-*Hras/p53* ^*-/-*^ and MMTV-*Hras/p53* ^*R172H/R172H*^ tumors continued to grow with unaltered rates for 3–4 days, after which the growth of most of the tumors began to plateau. Although the average size of the MMTV-*Hras/p53* ^*R172H/R172H*^ tumors at the initiation of treatment was larger than that of the MMTV-*Hras/p53* ^*-/-*^ tumors, the overall shape of the tumor response curves was nearly superimposable. Interestingly, 3 of the 18 MMTV-*Hras/p53* ^*R172H/R172H*^ tumors in the treatment study exhibited no plateau phase and continued to grow at an unaltered rate during the 9-day treatment period (S4 Fig.).

### Gene expression profiling of tumors with different p53 status

**Fig 6:**
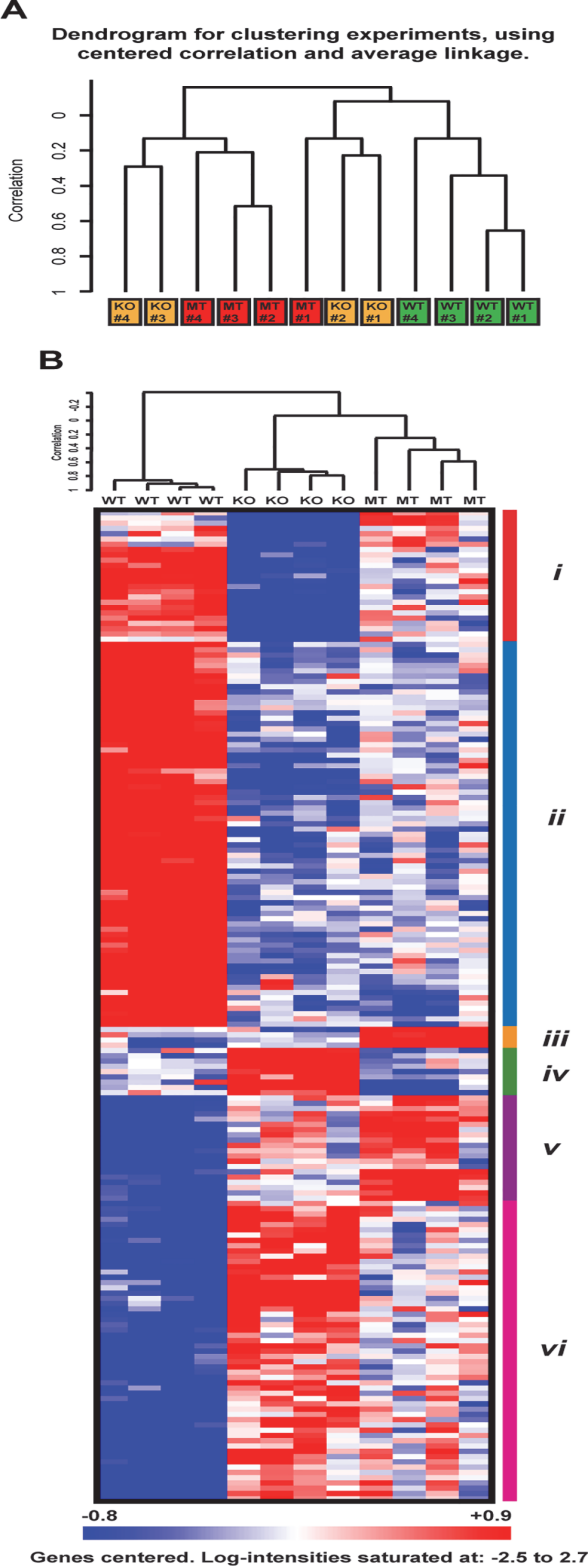
Gene expression profiling analysis of salivary tumors with different p53 status. (A) Unsupervised hierarchical clustering of MMTV-*Hras/p53* ^*+/+*^ (green), MMTV-*Hras/p53* ^*-/-*^ (yellow), and MMTV-*Hras/p53* ^*R172H/R172H*^ (red) tumors based on gene expression profiling. (B) Heatmap of the identified 188 genes through the multi-class comparison function of SAM with a low 1% False Discovery Rate (FDR) and hierarchical clustering. Red represents higher expression levels and blue represents lower levels, while white represents intermediate levels. The 6 sub-clusters identified through visual inspection are marked by color bars on the right (*i-vi*).

To compare the gene expression profiles of tumors with different p53 genotypic status and identify genes differentially regulated, 4 tumors from each genotypic group were analyzed using Affymetrix GeneChip oligonucleotide arrays. After the model-based expression summaries were obtained, an unsupervised hierarchical clustering analysis of the 12 tumor samples was conducted and is summarized in Fig. 6A. As expected, the 4 samples with wild-type p53 fell into a tight cluster, which indicates close similarity within the gene expression profiles of this group. On the other hand, this unsupervised clustering method failed to separate the MMTV-*Hras/p53* ^*-/-*^ or MMTV-*Hras/p53* ^*R172H/R172H*^ tumors into distinct groups, signifying the lack of significant numbers of genes that are consistently differentially expressed between the p53-null and mutant p53 tumors. This suggests that the primary effect of the R172H mutation is the disruption the DNA-binding domain and subsequent gene-regulation capacity of wild-type p53.

As our major interest was to identify differentially expressed genes that may indicate gain-of-function effects, we compared the expression profiles of the three groups of tumors, and tumors with wild-type p53 were used to control for residual wild-type functions of mutant p53. To identify the genes differentially expressed between the three groups, we performed class comparison analysis using Significance Analysis of Microarray (SAM). The multi-class comparison function of SAM gave rise to the identification of 188 genes with a low 1% False Discovery Rate (FDR). The 188 genes were then subjected to hierarchical clustering to identify co-expressed gene clusters, the result of which is shown in Fig. 6B. By visually examining the pattern of the expression levels across the three genotypic groups, the 188 genes were further categorized to 6 groups (*i* to *vi*). The expression levels and potential meaning of the 6 clusters of genes are summarized in S2 Table and the 188 genes are listed in S3 Table. Of the 188 genes, 184 show a clear difference in expression levels between samples with wild-type p53 and without p53 (except the 4 genes in Cluster *iii*), which represent the differentially expressed genes between the wild-type p53 (WT) and p53-null status in this analysis. Of these 184 genes, only a few were previously reported as regulated by wild-type p53, including 3 well-characterized p53 target genes *Cdkn1a (p21)*, *Mdm2*, and *Ccng1*. These genes are listed in S4 Table. To explore potential biological pathways in which these differentially expressed genes may function, this 184 gene-signature was submitted to Ingenuity Pathway Analysis, which identifies the most significantly perturbed signaling and metabolic pathways, molecular networks, and biological processes. The most highly enriched biological functions from the analysis are summarized in S5 Table. The top two networks identified are displayed in S5 Fig. The first network involves biological functions including Cancer, Cell Cycle, and Developmental Disorder, with a score of 37 defined by IPA and 20 focus molecules, where p53 sits right at the center. The second network is enriched for biological functions including Cell Death, Dermatological Diseases and Conditions, and Cancer, with a score of 34 and 19 focus molecules.

Of the 6 gene clusters, Cluster *iii* and *v* define the genes with higher expression levels in the mutant p53-expressing samples than in the wild-type p53-expressing and p53-null samples (S2 Table), which could potentially represent genes contributing to the gain-of-function properties of the p53 R172H mutant. As many of these genes have anti-apoptotic and cell migration-promoting activities (see DISCUSSION), these two clusters represent good candidates for the gain-of-function properties of the p53 R172H mutant, including insensitivity to treatment-induced cell death and elevated metastatic potential. Mutant p53-expressing samples retained expression levels similar to those samples with wild-type p53 (Cluster *iv*), or intermediate levels (Cluster *i* and *vi*) for a significant fraction of the remaining genes. This indicates the presence of residual wild-type functions with the mutant p53 in regulating gene expression. However, when examining the overall expression profiles based on these 188 genes, the two groups showing the greatest similarity were the mutant p53-expressing tumors and p53-null tumors, while both showed significant differences from the tumors with wild-type p53, in agreement with the results from the unsupervised clustering of the samples (Fig. 6A). To further support this notion, we performed class comparison analysis between MT-p53 and p53-null samples, as well as between wild-type p53 and p53-null samples, using three widely-used probe-level data-processing algorithms (RMA, MBEI, and MAS5) and two False Discovery Rate (FDR)-based statistical approaches (SAM and student *t*-test with Benjamini-Hochberg correction). From this analysis we found that despite the different scales of significant genes identified by different mathematical and statistical approaches, there are consistently very few genes standing out from the comparisons between MT-p53 and p53-null samples, in sharp contrast to those between wild-type p53 and p53-null samples (S6 Table). This demonstrates that in this MMTV-*Hras* mouse salivary tumor model, the p53^R172H^ mutant status shares a very similar gene expression profile with p53-null status.

**Fig 7:**
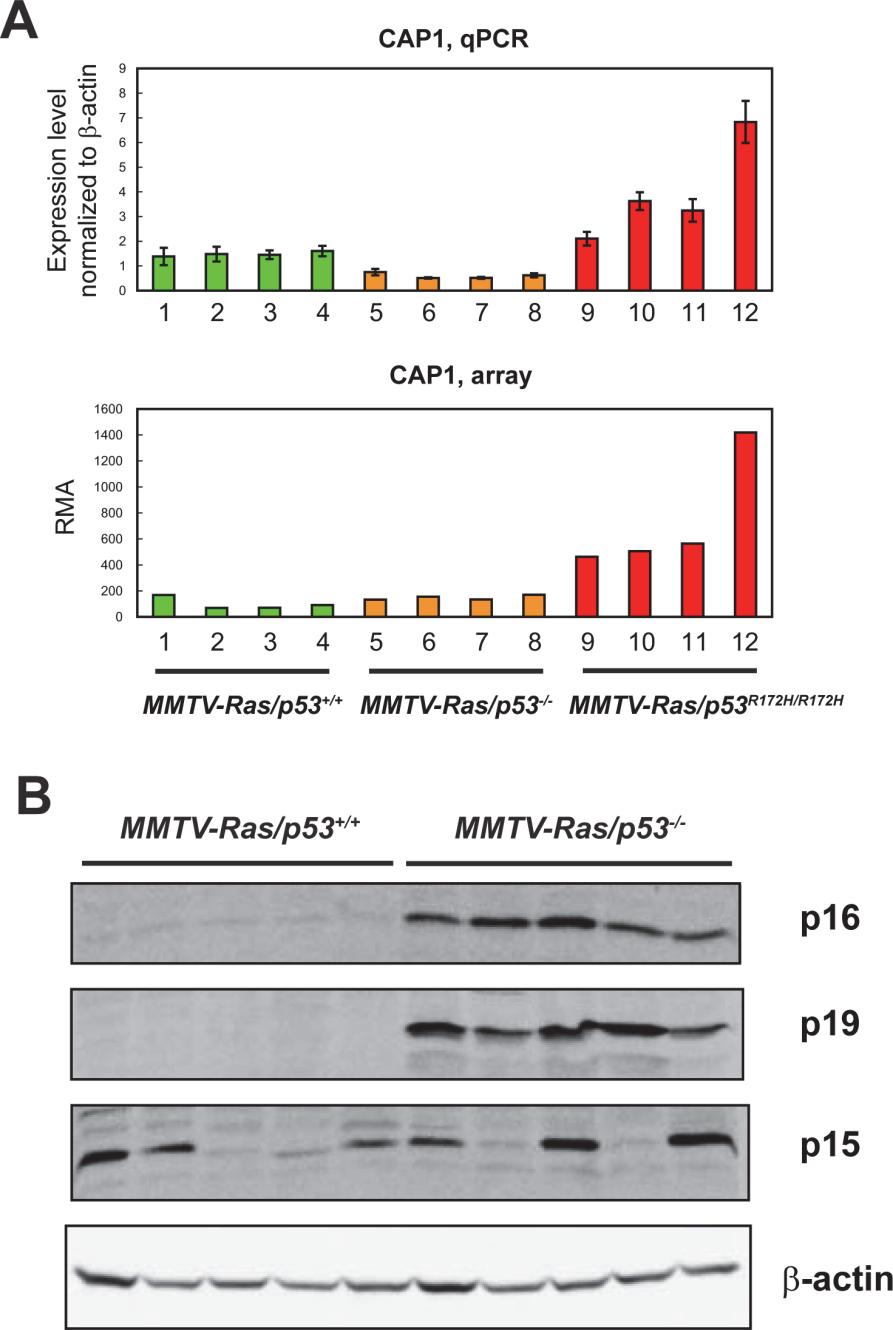
Validation of the microarray data for sample genes by qPCR and western blotting. (A) Top: Expression levels of the *Cap1* gene in 4 salivary tumors per group measured by qPCR normalized to β-actin ± coefficient of variance (top), and by microarray analysis (bottom). (B) Protein levels of p16^Ink4a^, p19^ARF^, and p15^Ink4b^ in 5 MMTV-*Hras/p53* ^*+/+*^ and 5 MMTV-*Hras/p53* ^*-/-*^ tumors. β-actin was used as a loading control.

To validate the microarray experiment, we performed real-time PCR to quantitate the expression levels of *Cap1*, which is significantly up-regulated in MT-p53 expressing samples compared to the other two groups of tumors. We also checked the protein levels of p16Ink4a and p19Arf, the two genes expressed from the *Cdkn2a* locus, which is down-regulated in wild-type p53-expressing samples based on the array analysis. Both the real-time PCR and Western Blotting analyses showed similar results as measured by microarray, which confirmed the validity of the microarray analysis (Fig. 7).

## Discussion

Beyond loss of p53 wild-type functions, a subset of p53 mutants gain novel functions promoting more severe malignant properties. Although extensive studies on the gain-of-function effects of mutant p53 have been carried out using *in vitro* cell culture systems, fewer *in vivo* models have been developed to investigate these effects under physiological conditions and in a normal cellular context with endogenous cell-cell and cell-ECM interactions. Our study contributes to research on the gain-of-function effects of mutant p53 by introducing a mutant p53 allele with well-characterized gain-of-function properties into the MMTV-*Hras* transgenic mouse model, in which deficiency for p53 has been shown to accelerate salivary tumorigenesis and alter tumor properties.

Interestingly, the data from this study demonstrated that MMTV-*Hras/p53* ^*R172H/R172H*^ and MMTV-*Hras/p53* ^*-/-*^ mice are very similar with regard to age of salivary tumor onset, tumor growth rates, tumor histopathological features, and response to a DNA-damaging agent, doxorubicin. This similarity is not due to lack of mutant p53 expression in the MMTV-*Hras/p53* ^*R172H/R172H*^ salivary tumors, because both western blotting and p53 immunohistochemistry analysis showed that mutant p53 accumulated to uniformly high levels in the tumors (Fig. 3). Compared to other *in vivo* analyses on the same p53 mutant through knock-in approach, our finding is not surprising. First, in a K-*ras*-driven lung adenocarcinoma model, neither the *p53* ^*R172H*^ nor the *p53* ^*R270H*^ mutant displayed any detectable gain-of-function activity compared to total p53 loss [49]. In another study, *WAP-Cre*-induced expression of the *p53* ^*R270H*^ mutant in p53-null mouse mammary glands caused no difference in tumor latency compared to p53-null glands [50]. These observations suggest that tissue specificity plays a role in the *in vivo* activity of these mutants. In addition, knock-in mouse models homozygous for the *p53* ^*R172H*^ mutation (or one *p53* ^*R172H*^ allele and one null allele) displayed no significant difference in survival time of the mice when compared to p53-null mice [43,44,51]. One exception was a model expressing a humanized *p53* ^*R248Q*^ allele, which significantly reduced lifespan when compared to p53-null mice [52]. In two of the studies [44,51], there was no difference between the tumor spectra of *p53* ^*R172H/R172H*^ and *p53* ^*-/-*^ mice, while in the third study [43], *p53* ^*R172H/-*^ mice developed carcinomas and hemangiosarcomas with increased incidence compared to *p53* ^*-/-*^ mice. The discrepancy between these studies may be due to the fact that mice with different genetic backgrounds were used. The common gain-of-function effect observed in all three studies was that tumors in the *p53* ^*R172H/+*^ mice metastasized with a high incidence, a feature the *p53* ^*+/-*^ tumors lacked.

In this study, with a set of well-defined histopathologic parameters, the MMTV-*Hras/p53* ^*-/-*^ tumors displayed more malignant properties than the MMTV-*Hras/p53* ^*+/+*^ tumors, consistent with our previous findings [46]. The MMTV-*Hras/p53* ^*R172H/R172H*^ tumors closely resemble the MMTV-*Hras/p53* ^*-/-*^ tumors with regard to most of these parameters, suggesting that loss of the sequence-specific DNA binding function of wild-type p53 is the primary cause of the difference in tumor histology between tumors with mutant and wild-type p53.

In a previous study from our lab [47], the MMTV-*Hras/p53* ^*-/-*^ tumors displayed a delayed response upon doxorubicin treatment, as compared to the MMTV-*Hras/p53* ^*+/+*^ tumors. Apoptotic levels were shown to be minimal in both types of tumors, either with or without treatment. Instead, doxorubicin treatment caused significant changes in cell cycle distribution in these tumor cells. In the present study, the MMTV-*Hras/p53* ^*R172H/R172H*^ tumors demonstrated indistinguishable response patterns compared to the MMTV-*Hras/p53* ^*-/-*^ tumors. Immunohistochemical analysis of cleaved caspase 3 (Cc3) revealed similarly low levels of apoptosis in tumors of all three genotypes (data not shown). Thus, the results demonstrated again that apoptosis does not play a major role in tumor properties or response to chemotherapeutic treatments in this model, likely due to the anti-apoptotic effects of the activated Ras signaling driving tumorigenesis in this model. Thus, it is important to note that, with the low level of both spontaneous and drug-inducible apoptosis in this tumor model, it may be difficult to detect any influence of mutant p53 on apoptosis, which is an important aspect of the gain-of-function activities identified in *in vitro* cell culture systems.

One possible explanation for the failure to detect a p53 gain-of-function phenotype distinct from the knock-out phenotype could potentially be a lack of stabilization and accumulation of the mutant p53 protein. The Lozano group demonstrated that in the R172H knock-in mice, mutant p53 was not stabilized in either normal or tumor tissues, due to the basal level of MDM2’s inhibitory activity on mutant p53 [53]. They also demonstrated that various types of stresses that stabilize wild-type p53 also stabilize p53^R172H^ [54]. However, the apparent absence of a gain-of-function phenotype in our study is not likely due to the stability issue, because mutant p53^R172H^ was found to be stabilized in all the tumors tested by both Western blotting and immunohistochemistry. It is reasonable to speculate that activated Ras signaling is providing the stabilizing signal for mutant p53 in this tumor model.

Given the similarity between the p53 R172H-expressing and p53-null tumors, but the significant difference between both groups and tumors with wild-type p53 in the *in vivo* analyses we performed, it is not surprising that the gene expression profiles of the different groups of tumors closely reflect this relationship. We have tested three prevalent probe-level data summarization algorithms for Affymetrix microarray data, and two False Discovery Rate-based class comparison approaches, and none of the combinations could detect reasonable numbers of significant genes from the comparison between the MMTV-*Hras/p53* ^*-/-*^ and MMTV-*Hras/p53* ^*R172H/R172H*^ tumors, while substantial numbers of genes were identified between the MMTV-*Hras/p53* ^*+/+*^ tumors and either the MMTV-*Hras/p53* ^*-/-*^ or the MMTV-*Hras/p53* ^*R172H/R172H*^ tumors.

Several mechanisms have been proposed for the gain-of-function activities of mutant p53 [38]. One of these mechanisms proposes that mutant p53 actively regulates a unique set of genes, the activities of which endow the cell with a growth advantage, chemoresistance, altered metabolism and other properties [38]. Unlike wild-type p53, which depends on its sequence-specific DNA binding for its transactivation activity, genes regulated by mutant p53 lack a consensus DNA binding site in their promoter regions [38]. It has thus been proposed that instead of binding to a common response sequence, mutant p53 preferentially binds to stereo-specific DNA configurations [55,56]. Alternatively, mutant p53 may bind to target genes indirectly, i.e. by interactions with other transcription factors, e.g. Sp1 [57,58], Ets [59], NF-Y [21], VDR [60] and SREBP [35]. Although these findings support a transcription-dependent mechanism for the gain-of-function activities of mutant p53, this hasn’t been tested strictly in an *in vivo* setting or under physiological conditions. Rather, most of the observations have been made by over-expressing mutant p53 in p53-null cells, and in many cases, in the presence of stress signals. Additionally, almost all of the previous studies only utilized a comparison between cells without p53 and those expressing mutant p53, a study design lacking the ability to detect any residual wild-type function of the mutants.

The class comparison analysis in this study identified a small subset of genes with the highest expression levels in the MMTV-*Hras/p53* ^*R172H/R172H*^ tumors (Cluster *iii* and *v* in Fig. 6; S2 Table). We also compared our microarray results to those from other gene expression profiling analyses designed to identify mutant p53-regulated genes, either from cells cultured *in vitro* [18,61–63] or tumors from *in vivo* mouse models [64]. One of the mutant p53-induced genes we identified, *Bcl2l1* (Bcl2-like 1, also known as *Bclx* or *BCL-X(L)* in human) was previously identified by another group as induced by p53R 175H overexpressed in p53-null H1299 non-small cell lung carcinoma cells. BCL2L1 is a member of the BCL2 family of anti-apoptotic proteins [65]. In addition, expression of *BCL2L1* was dependent on mutant p53 in SKBR3 mammary adenocarcinoma cells expressing p53R 175H, and in HT29 human colorectal adenocarcinoma cells expressing p53 R273H, another p53 mutant with gain-of-function activities [63]. Another gene found to be most highly expressed in the MMTV-*Hras/p53* ^*R172H/R172H*^ tumors, *Csnk2a1*, which encodes casein kinase 2, alpha 1 polypeptide, has also been shown to have an anti-apoptotic function, but regulation of its expression hasn’t been associated with mutant p53[66–68]).

Because increased metastatic potential has also been commonly observed with gain-of-function mutants of p53 [43,44], and p53 R175H was shown to promote *in vitro* cell migration as well as *in vivo* tumor metastasis [44], we looked for genes that may contribute to these activities among the list of genes most highly expressed in the MMTV-*Hras/p53* ^*R172H/R172H*^ tumors. Indeed, a few genes have been reported to promote cell motility and/or metastasis, including *Etv4/E1A-F* [69,70], *Mia1* [71], and *Cap1* [72]. Another gene with previously identified association with p53 R175H, *EFEMP2* (EGF-containing fibulin-like extracellular matrix protein 2) was recognized as a binding partner of mutant human p53 proteins, with the highest specificity to structural mutants like human p53^R175H^. Even more intriguingly, co-expression of EFEMP2 and p53 R175H had synergistic effects in promoting neoplastic transformation and tumor cell growth [73]. The fact that we didn’t observe a significant decrease in apoptotic levels or response to doxorubicin treatment, and we didn't identify any obvious elevated metastatic potential of the p53 R172H-expressing tumors, as compared to p53-null tumors, suggests that these gain-of-function properties may be cell/tumor type-specific and depend on specific cellular contexts and molecular events.

It should be noted that although mutant p53 R172H loses its sequence-specific DNA binding activity, it still maintains intact N-terminal and C-terminal domains. Thus, an additional proposed mechanism for the gain-of-function effects of mutant p53 involves the retention of some residual wild-type function, which when imbalanced and deregulated may contribute to opposite effects [40]. In this study, gene expression profiling also identified a group of genes similarly regulated in the MMTV-*Hras/p53* ^*+/+*^ and MMTV-*Hras/p53* ^*R172H/R172H*^ tumors as compared to the MMTV-*Hras/p53* ^*-/-*^ tumors (Cluster *iv* and *vi* in Fig. 6; S2 Table). Although overall the expression profiles of the MMTV-*Hras/p53* ^*R172H/R172H*^ and MMTV-*Hras/p53* ^*-/-*^ tumors are very similar to each other (S6 Table), the small subset of genes co-regulated in tumors with wild-type and mutant p53 might help elucidate pathways shared by these two functionally very different proteins, and provide insights into the mechanistic basis of the gain-of-function effects.

In conclusion, this study has led to the generation of MMTV-*Hras/p53* ^*R172H/R172H*^ mice and a comparison between these mice and the previously characterized MMTV-*Hras/p53* ^*+/+*^ and MMTV-*Hras/p53* ^*-/-*^ mice, with regard to salivary tumorigenesis, tumor properties, tumor responses to a chemotherapeutic agent, and tumor gene expression profiles. This study enabled us to compare the influences of different p53 status (wild-type, null, and mutant) on *in vivo* tumor development and test the gain-of-function effects of a p53 mutant in an *in vivo* setting. Overall, the MMTV-*Hras/p53* ^*R172H/R172H*^ mice closely resembled the MMTV-*Hras/p53* ^*-/-*^ mice with regard to age of tumor onset, tumor growth rates, tumor histopathological properties, tumor responses to doxorubicin, and gene expression profiles, while both groups were clearly distinct from the MMTV-*Hras/p53* ^*+/+*^ mice. These results indicate that in this MMTV-*Hras*-driven mouse salivary tumor model, the primary effect of p53 R172H mutation is the loss of wild-type p53 function, with little or no gain-of-function effect on tumorigenesis, underscoring the tissue- and tumor type-specific properties of the gain-of-function mutants of p53.

## Supporting Information

S1 Fig Tumor growth rates. (A-C) Once a tumor was detected, growth was monitored by daily caliper measurements, and calculated tumor weights (mg) of the three groups of tumors were plotted over time. Each line represents the growth of an individual tumor.
(TIF)

S2 Fig Tumor histopathology. Tumor histopathology was graded on a 3-point scale taking into account (A) N/C (nuclear to cytoplasmic) ratio, degree of nuclear pleomorphism, and overall tumor architecture; percentage of tumor cells showing (B) spindle cell morphology, (C) “giant cells”, (D) apoptosis, (E) mitotic figures, and (F) necrosis.
(TIF)

S3 Fig Ki-67 staining. (A) Heterogeneity of Ki-67 staining in different tumors from the same genotypic group. Top: MMTV-*Hras/p53* ^*+/+*^; middle: MMTV-*Hras/p53* ^*-/-*^; bottom: MMTV-*Hras/p53* ^*R172H/R172H*^; left: representative tumors of each genotype with high levels of Ki-67 staining; right: representative tumors of each genotype with low levels of Ki-67 staining. (B) An example of an MMTV-*Hras/p53* ^*+/+*^ tumor in which different regions show markedly different levels of Ki-67 staining.
(TIF)

S4 Fig Tumor growth responses to doxorubicin. . (A-C) Calculated weights (mg) of the three groups of tumors during the treatment period are plotted over time (days). Each line represents the growth of an individual tumor.
(TIF)

S5 Fig The top two networks identified through Ingenuity Pathway Analysis (IPA) on the significantly differentially expressed genes. Genes up-regulated in wild-type p53 tumors compared to p53-null tumors are colored in red and those down-regulated are colored in green.
(TIF)

S1 Table Summary of the mice used for the age of tumor onset analysis (DOCX)

S2 Table Overall expression levels and functional meaning of genes in different clusters (DOCX)

S3 Table List of genes in different clusters (DOCX)

S4 Table Genes previously reported as regulated by p53 (DOCX)

S5 Table Top enriched biological functions from WT-p53 vs. p53-null comparison (DOCX)

S6 Table Number of significant genes from different probe-level data summarization algorithms and statistical approaches (DOCX)

S7 Table Primers used in the quantitative PCR assays (DOCX)

S1 Methods Supplementary Materials and Methods (DOCX)
