# Supplementary figures and images for: Comparison of Effects of p53 Null and Gain-of-Function Mutations on Salivary Tumors in MMTV-Hras Transgenic Mice
Source: PLoS One. 2015 Feb 19;10(2):e0118029. doi: 10.1371/journal.pone.0118029 (PMC4335025; doi:10.1371/journal.pone.0118029)

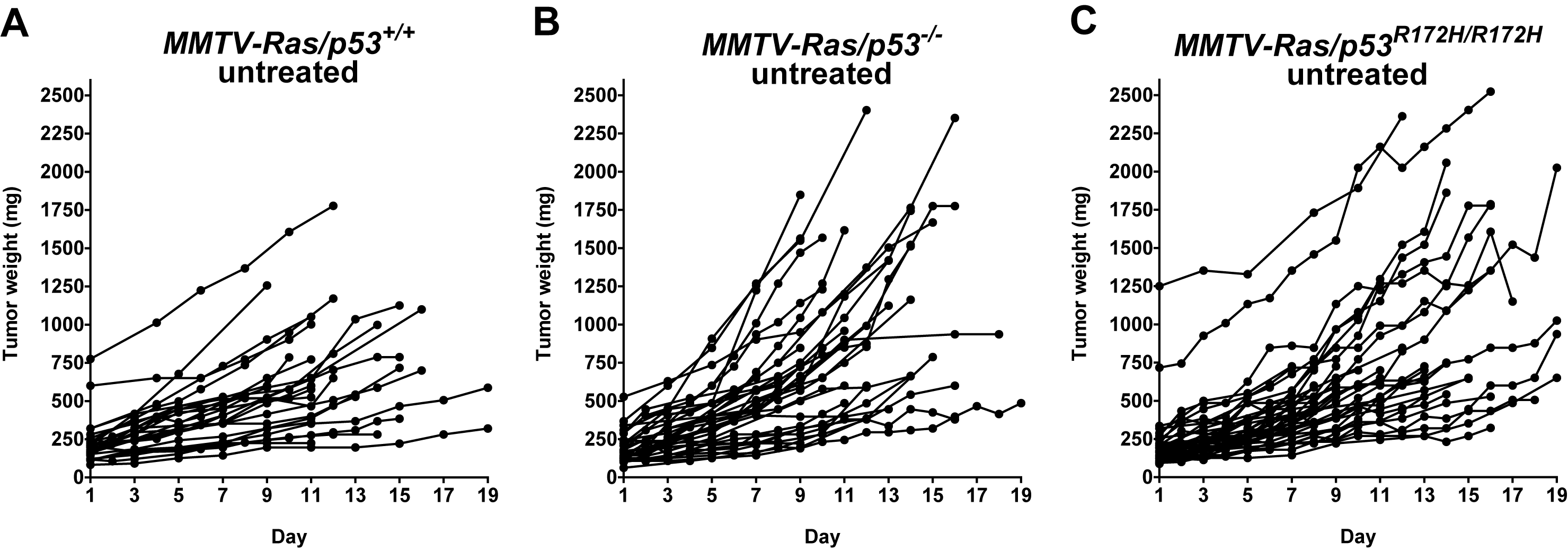

Supplement: S1 Fig — (A-C) Once a tumor was detected, growth was monitored by daily caliper measurements, and calculated tumor weights (mg) of the three groups of tumors were plotted over time. Each line represents the growth of an individual tumor. (TIF) [file pone.0118029.s001.tif]

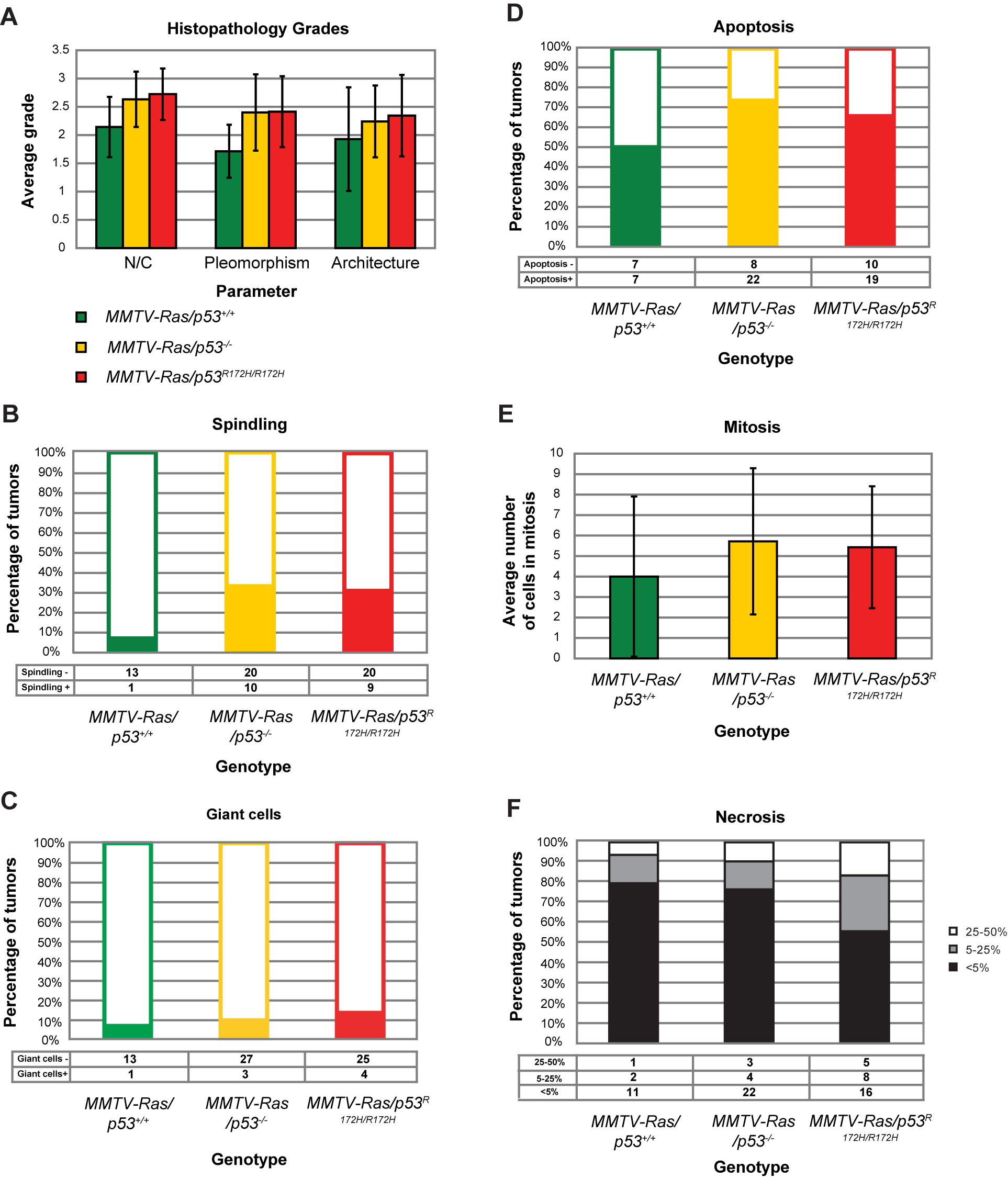

Supplement: S2 Fig — Tumor histopathology was graded on a 3-point scale taking into account (A) N/C (nuclear to cytoplasmic) ratio, degree of nuclear pleomorphism, and overall tumor architecture; percentage of tumor cells showing (B) spindle cell morphology, (C) “giant cells”, (D) apoptosis, (E) mitotic figures, and (F) necrosis. (TIF) [file pone.0118029.s002.tif]

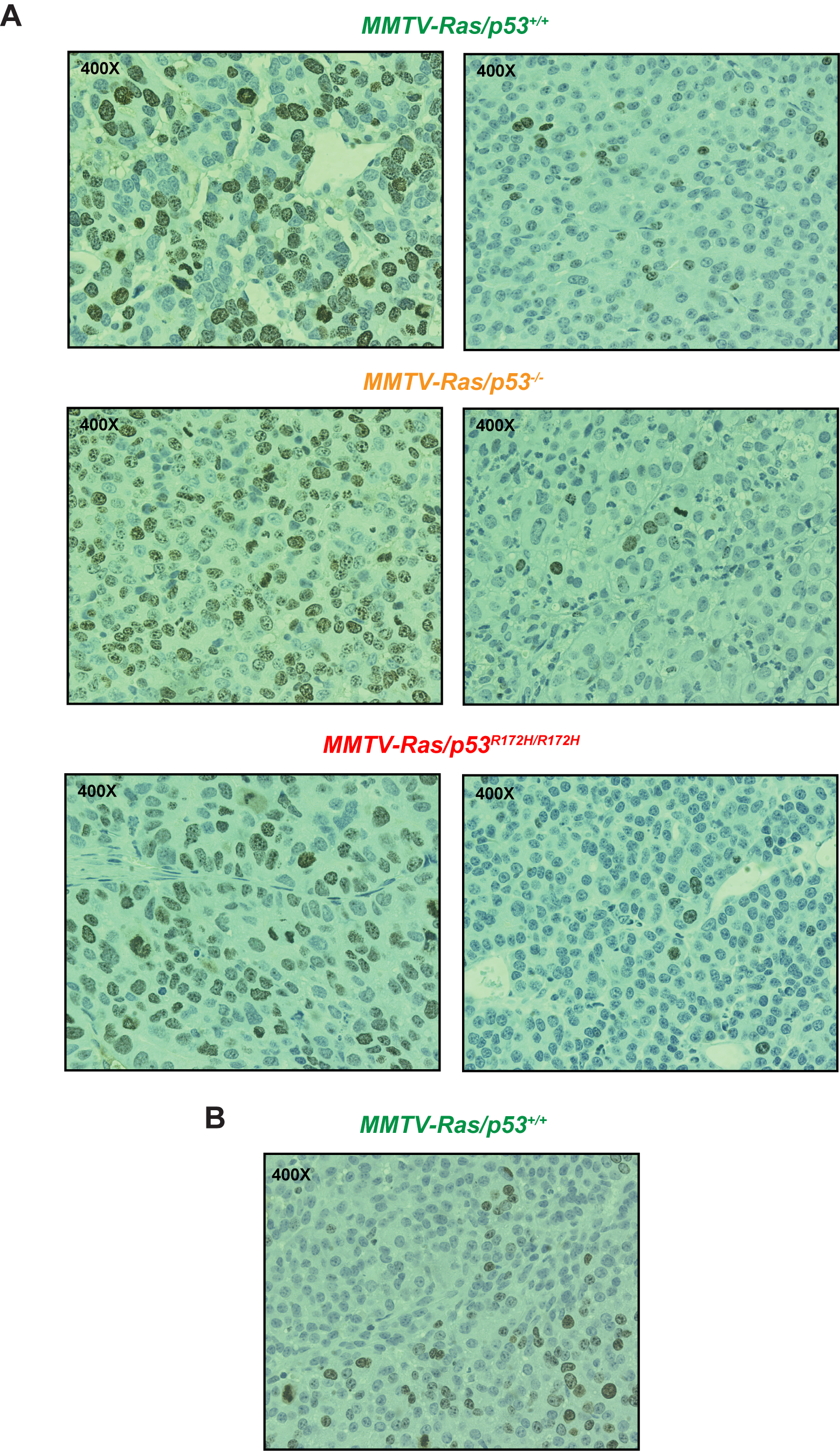

Supplement: S3 Fig — (A) Heterogeneity of Ki-67 staining in different tumors from the same genotypic group. Top: MMTV-Hras/p53 +/+; middle: MMTV-Hras/p53 -/-; bottom: MMTV-Hras/p53 R172H/R172H; left: representative tumors of each genotype with high levels of Ki-67 staining; right: representative tumors of each genotype with low levels of Ki-67 staining. (B) An example of an MMTV-Hras/p53 +/+ tumor in which different regions show markedly different levels of Ki-67 staining. (TIF) [file pone.0118029.s003.tif]

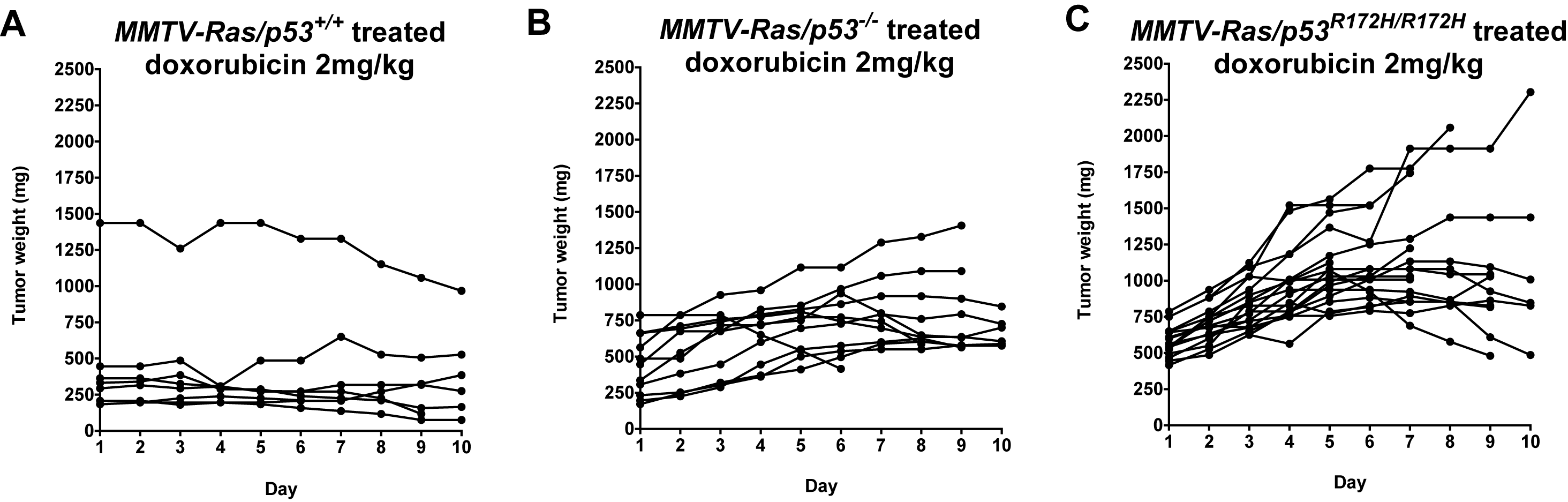

Supplement: S4 Fig — . (A-C) Calculated weights (mg) of the three groups of tumors during the treatment period are plotted over time (days). Each line represents the growth of an individual tumor. (TIF) [file pone.0118029.s004.tif]

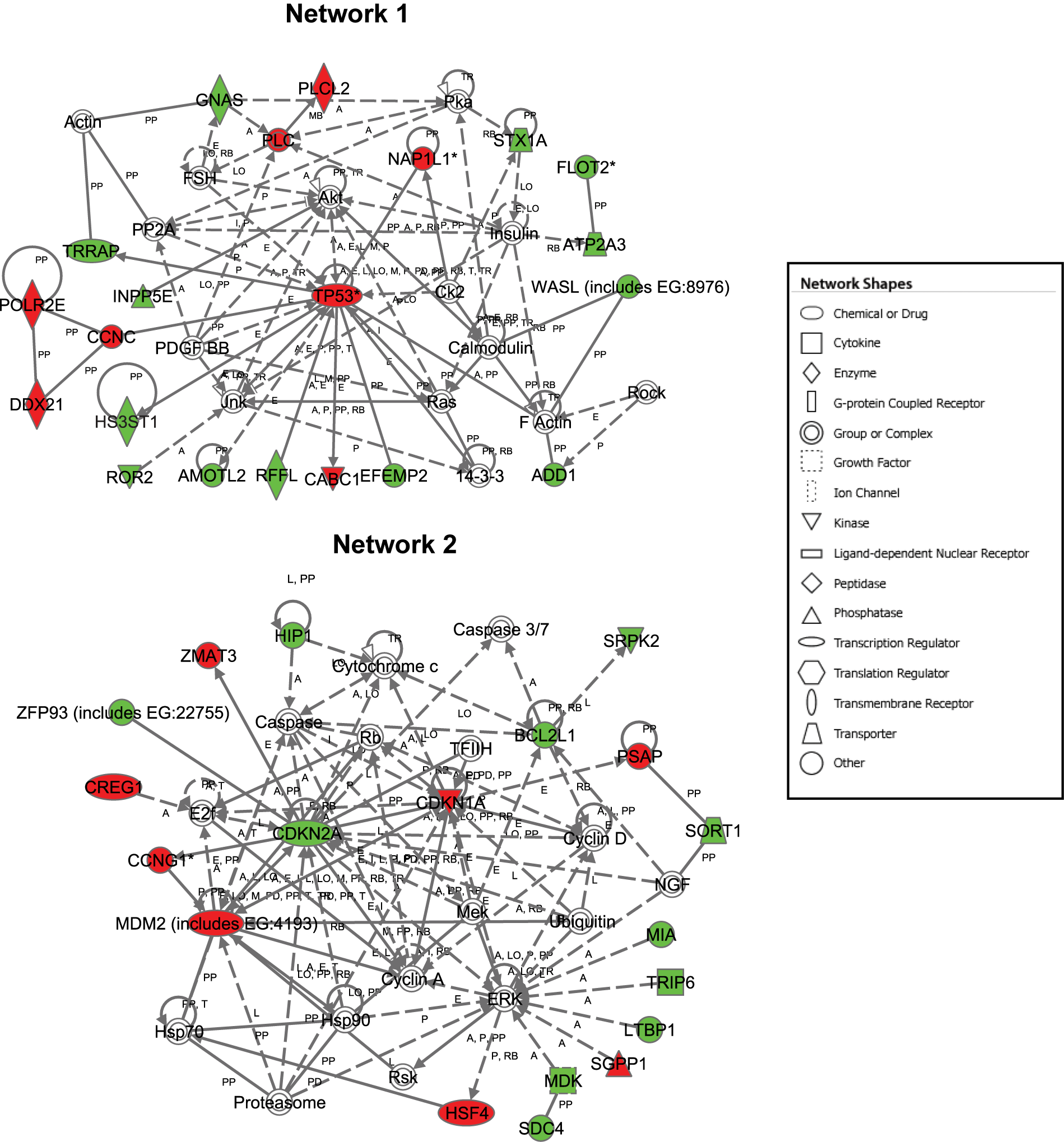

Supplement: S5 Fig — Genes up-regulated in wild-type p53 tumors compared to p53-null tumors are colored in red and those down-regulated are colored in green. (TIF) [file pone.0118029.s005.tif]
