# Supplementary material for: Comparison of Effects of p53 Null and Gain-of-Function Mutations on Salivary Tumors in MMTV-Hras Transgenic Mice
Source: PLoS One. 2015 Feb 19;10(2):e0118029. doi: 10.1371/journal.pone.0118029 (PMC4335025; doi:10.1371/journal.pone.0118029)
Supplement: S1 Table — (DOCX) [file pone.0118029.s006.docx]

**S1 Table. Summary of the mice used for the age of tumor onset analysis***

| **Genotype** | ***MMTV-Ras/p53^+/+^*** | ***MMTV-Ras/p53^-/-^*** | ***MMTV-Ras/p53^R172H/R172H^*** |
| --- | --- | --- | --- |
| # of males | 114 | 138 | 134 |
| # of males with salivary tumor | 11 | 52 | 66 |
| Average age of onset (days) | 234 | 89 | 114 |
| Median age of onset (days) | 177 | 68 | 109 |
| Died w/o salivary tumor | 38 | 86 | 68 |
| Alive | 65 | 0 | 0 |

* The study was followed up for 479 days for the *ras/p53^+/+^* mouse that developed salivary tumors the latest.
