# Supplementary material for: Comparison of Effects of p53 Null and Gain-of-Function Mutations on Salivary Tumors in MMTV-Hras Transgenic Mice
Source: PLoS One. 2015 Feb 19;10(2):e0118029. doi: 10.1371/journal.pone.0118029 (PMC4335025; doi:10.1371/journal.pone.0118029)
Supplement: S2 Table — (DOCX) [file pone.0118029.s007.docx]

**S2 Table. Overall expression levels and functional meaning of genes in different clusters**

| **Cluster** | **# of genes** | **Overall expression level** | | | **Potential Meaning for MT** |
| --- | --- | --- | --- | --- | --- |
|  |  | WT | KO | MT |  |
| i | 7 | medium | low | high | gain-of-function/residual function |
| ii | 91 | high | low | low | loss-of-function |
| iii | 4 | low | low | high | gain-of-function |
| iv | 9 | low | high | low | residual function |
| v | 21 | low | medium | high | gain-of-function |
| vi | 56 | low | high | medium | residual function |
