# Supplementary material for: Comparison of Effects of p53 Null and Gain-of-Function Mutations on Salivary Tumors in MMTV-Hras Transgenic Mice
Source: PLoS One. 2015 Feb 19;10(2):e0118029. doi: 10.1371/journal.pone.0118029 (PMC4335025; doi:10.1371/journal.pone.0118029)
Supplement: S3 Table — (DOCX) [file pone.0118029.s008.docx]

**S3 Table. List of genes in different clusters**

| **Affymetrix ID** | **Accession** | **Gene Symble** | **Gene Name** |
| --- | --- | --- | --- |
| **Cluster *i*** |  |  |  |
| 1415677_at | NM_026819 | Dhrs1 | dehydrogenase/reductase (SDR family) member 1 |
| 1420986_s_at | BB448784 | Kif3b | kinesin family member 3B |
| 1421813_a_at | BM212050 | Psap | prosaposin |
| 1423617_at | AA790871 | Cog8 | component of oligomeric golgi complex 8 |
| 1426538_a_at | BB828014 | Trp53 | transformation related protein 53 |
| 1427739_a_at | AJ297973 | Trp53 | transformation related protein 53 |
| 1450007_at | NM_019769 | Chp | calcium-binding protein P22 |
| **Cluster *ii*** |  |  |  |
| 1415947_at | BC027426 | Creg1 | cellular repressor of E1A-stimulated genes 1 |
| 1415948_at | BC027426 | Creg1 | cellular repressor of E1A-stimulated genes 1 |
| 1416052_at | AK011304 | Prps1 | phosphoribosyl pyrophosphate synthetase 1 |
| 1416635_at | NM_020561 | Smpdl3a | sphingomyelin phosphodiesterase, acid-like 3A |
| 1416795_at | NM_030004 | Cryl1 | crystallin, lambda 1 |
| 1416811_s_at | NM_007796 | Ctla2a /// Ctla2b | cytotoxic T lymphocyte-associated protein 2 alpha /// cytotoxic T lymphocyte-associated protein 2 beta |
| 1416849_at | NM_134007 | Cisd1 | CDGSH iron sulfur domain 1 |
| 1416850_s_at | NM_134007 | Cisd1 | CDGSH iron sulfur domain 1 |
| 1416930_at | NM_010742 | Ly6d | lymphocyte antigen 6 complex, locus D |
| 1417066_at | AK014605 | Cabc1 | chaperone, ABC1 activity of bc1 complex like (S. pombe) |
| 1417138_s_at | NM_025554 | Polr2e | polymerase (RNA) II (DNA directed) polypeptide E |
| 1417459_at | NM_019910 | Dcpp1 | demilune cell and parotid protein 1 |
| 1417696_at | BG064396 | Soat1 | sterol O-acyltransferase 1 |
| 1417697_at | BG064396 | Soat1 | sterol O-acyltransferase 1 |
| 1417837_at | NM_009434 | Phlda2 | pleckstrin homology-like domain, family A, member 2 |
| 1418165_at | NM_010584 | Itln1 | intelectin 1 (galactofuranose binding) |
| 1420476_a_at | BG064031 | Nap1l1 | nucleosome assembly protein 1-like 1 /// similar to nucleosome assembly protein 1-like 1 |
| 1420478_at | BG064031 | Nap1l1 | nucleosome assembly protein 1-like 1 |
| 1420532_at | NM_015823 | Magi2 | membrane associated guanylate kinase, WW and PDZ domain containing 2 |
| 1420827_a_at | BG065754 | Ccng1 | cyclin G1 |
| 1421490_at | NM_019628 | Prpmp5 | proline-rich protein MP5 |
| 1421708_a_at | NM_009284 | Stat6 | signal transducer and activator of transcription 6 |
| 1422577_at | AB056479 | Cs | citrate synthase |
| 1422875_at | NM_013489 | Cd84 | CD84 antigen |
| 1423610_at | BG086961 | Metap2 | methionine aminopeptidase 2 |
| 1423842_a_at | AF305730 | Rnf41 | ring finger protein 41 |
| 1423957_at | BI440638 | Isg20l1 | interferon stimulated exonuclease gene 20-like 1 |
| 1424265_at | BC022734 | Npl | N-acetylneuraminate pyruvate lyase |
| 1424487_x_at | BB284199 | Txnrd1 | thioredoxin reductase 1 |
| 1424638_at | AK007630 | Cdkn1a | cyclin-dependent kinase inhibitor 1A (P21) |
| 1425786_a_at | AF160966 | Hsf4 | heat shock transcription factor 4 |
| 1426450_at | BM207017 | Plcl2 | phospholipase C-like 2 |
| 1426646_at | AK018610 | 9130011J15Rik | RIKEN cDNA 9130011J15 gene |
| 1426813_at | U01139 | Ltv1 | LTV1 homolog (S. cerevisiae) |
| 1426936_at | BC002257 | LOC629242 | hypothetical protein LOC629242 |
| 1426975_at | BG067859 | Os9 | amplified in osteosarcoma |
| 1427718_a_at | X58876 | Mdm2 | transformed mouse 3T3 cell double minute 2 |
| 1427745_x_at | X63005 | Prpmp5 |  |
| 1427862_at | AF133910 | Arl6ip3 | ADP-ribosylation factor-like 6 interacting protein 3 |
| 1428954_at | AK004710 | Slc9a3r2 | solute carrier family 9 (sodium/hydrogen exchanger), isoform 3 regulator 2 |
| 1429227_x_at | AK007322 | Nap1l1 | nucleosome assembly protein 1-like 1 |
| 1430869_a_at | BF460630 | Habp4 | hyaluronic acid binding protein 4 |
| 1431295_a_at | AK016910 | Stx18 | syntaxin 18 |
| 1432436_a_at | AK007618 | Ak3 | adenylate kinase 3 |
| 1433496_at | AV122321 | Glt25d1 | glycosyltransferase 25 domain containing 1 |
| 1433711_s_at | BG076140 | Sesn1 | sestrin 1 /// similar to Sesn1 protein |
| 1433720_s_at | AI647775 | Ndg2 | Nur77 downstream gene 2 |
| 1433783_at | BB479063 | Ldb3 | LIM domain binding 3 |
| 1434366_x_at | AW227993 | C1qb | complement component 1, q subcomponent, beta polypeptide |
| 1435335_a_at | BG144467 | Gnptab | N-acetylglucosamine-1-phosphate transferase, alpha and beta subunits |
| 1435628_x_at | AW763751 | LOC629242 | hypothetical protein LOC629242 |
| 1435685_x_at | AV150520 | Abcc5 | ATP-binding cassette, sub-family C (CFTR/MRP), member 5 |
| 1436097_x_at | BB327418 | Arhgap9 | Rho GTPase activating protein 9 |
| 1436198_at | BF468231 | 2310075K07Rik | RIKEN cDNA 2310075K07 gene |
| 1436990_s_at | AA038464 | Ndg2 | Nur77 downstream gene 2 |
| 1437401_at | BG075165 | Igf1 | insulin-like growth factor 1 |
| 1438199_at | AW228053 | AI316807 | expressed sequence AI316807 |
| 1438545_at | C81442 | Slc25a5 |  |
| 1438619_x_at | BB318221 | Zdhhc14 | zinc finger, DHHC domain containing 14 |
| 1448068_at | AI158425 | Soat1 | sterol O-acyltransferase 1 |
| 1448271_a_at | BM246099 | Ddx21 | DEAD (Asp-Glu-Ala-Asp) box polypeptide 21 |
| 1448303_at | NM_053110 | Gpnmb | glycoprotein (transmembrane) nmb |
| 1448508_at | NM_134000 | Traf3ip2 | Traf3 interacting protein 2 |
| 1448700_at | NM_008059 | G0s2 | G0/G1 switch gene 2 |
| 1448856_a_at | NM_026322 | Msra | methionine sulfoxide reductase A |
| 1449078_at | NM_018784 | St3gal6 | ST3 beta-galactoside alpha-2,3-sialyltransferase 6 |
| 1449079_s_at | NM_018784 | St3gal6 | ST3 beta-galactoside alpha-2,3-sialyltransferase 6 |
| 1449353_at | NM_009517 | Zmat3 | zinc finger matrin type 3 |
| 1449402_at | AB046929 | Chst7 | carbohydrate (N-acetylglucosamino) sulfotransferase 7 |
| 1450015_x_at | NM_030750 | Sgpp1 | sphingosine-1-phosphate phosphatase 1 |
| 1450016_at | BG065754 | Ccng1 | cyclin G1 |
| 1450017_at | BG065754 | Ccng1 | cyclin G1 |
| 1450667_a_at | AB056479 | Cs | citrate synthase |
| 1450714_at | BE626090 | Azin1 | antizyme inhibitor 1 |
| 1450883_a_at | BB534670 | Cd36 | CD36 antigen |
| 1451611_at | BC024581 | Hrasls3 | HRAS like suppressor 3 |
| 1451650_at | BC006690 | Ddo | D-aspartate oxidase |
| 1451768_a_at | AF196476 | Slc20a2 | solute carrier family 20, member 2 |
| 1451886_at | AF215896 | Speg | SPEG complex locus |
| 1451999_at | AF114378 | Ldb3 | LIM domain binding 3 |
| 1452014_a_at | AF440694 | Igf1 | insulin-like growth factor 1 |
| 1452778_x_at | AK004633 | Nap1l1 | nucleosome assembly protein 1-like 1 |
| 1453249_a_at | AK016448 | Tex21 | testis expressed gene 21 /// similar to tsec-2 |
| 1453604_a_at | AK012856 | Hbs1l | Hbs1-like (S. cerevisiae) |
| 1454142_a_at | AK011891 | Pwp1 | PWP1 homolog (S. cerevisiae) |
| 1454144_a_at | AK014079 | Ccnc | cyclin C |
| 1455765_a_at | BB515948 | Abcc8 | ATP-binding cassette, sub-family C (CFTR/MRP), member 8 |
| 1456319_at | BG065719 |  |  |
| 1460358_s_at | BC019768 | Nudt22 | nudix (nucleoside diphosphate linked moiety X)-type motif 22 |
| 1460652_at | NM_007953 | Esrra | estrogen related receptor, alpha |
| AFFX-18SRNAMur |  | Rn18s | 18S RNA |
| **Cluster *iii*** |  |  |  |
| 1417461_at | NM_007598 | Cap1 | CAP, adenylate cyclase-associated protein 1 (yeast) |
| 1417462_at | NM_007598 | Cap1 | CAP, adenylate cyclase-associated protein 1 (yeast) |
| 1435129_at | AW495875 |  | Transcribed locus |
| 1451477_at | AV047635 | OTTMUSG00000010657 | predicted gene, OTTMUSG00000010657 |
| **Cluster *iv*** |  |  |  |
| 1417761_at | BC010769 | Apoa4 | apolipoprotein A-IV |
| 1418694_at | BG071725 | Kcmf1 | potassium channel modulatory factor 1 |
| 1426865_a_at | BB698413 | Ncam1 | neural cell adhesion molecule 1 |
| 1436165_at | BI076494 | Luc7l2 | LUC7-like 2 (S. cerevisiae) |
| 1436504_x_at | AV027367 | Apoa4 | apolipoprotein A-IV |
| 1438167_x_at | AV269574 | Flcn | Folliculin |
| 1439040_at | BG068387 | Cenpe | centromere protein E |
| 1452193_a_at | BF466143 | Wasl | Wiskott-Aldrich syndrome-like (human) |
| 1456279_a_at | AV094648 | Bcap31 | B-cell receptor-associated protein 31 |
| **Cluster *v*** |  |  |  |
| 1416457_at | NM_016765 | Ddah2 | dimethylarginine dimethylaminohydrolase 2 |
| 1417018_at | NM_021474 | Efemp2 | epidermal growth factor-containing fibulin-like extracellular matrix protein 2 |
| 1417544_a_at | NM_008028 | Flot2 | flotillin 2 |
| 1418955_at | NM_009567 | Zfp93 | zinc finger protein 93 |
| 1419036_at | BB283759 | Csnk2a1 | casein kinase 2, alpha 1 polypeptide |
| 1419608_a_at | NM_019394 | Mia1 | melanoma inhibitory activity 1 |
| 1420887_a_at | NM_009743 | Bcl2l1 | Bcl2-like 1 |
| 1421090_at | NM_013510 | Epb4.1l1 | erythrocyte protein band 4.1-like 1 |
| 1423229_at | BM217803 | Inpp5e | inositol polyphosphate-5-phosphatase E |
| 1423232_at | X63190 | Etv4 | ets variant gene 4 (E1A enhancer binding protein, E1AF) |
| 1423363_at | AV247637 | Sort1 | sortilin 1 |
| 1427688_a_at | D28531 | Ptprs | protein tyrosine phosphatase, receptor type, S |
| 1427986_a_at | BB766878 | Col16a1 | collagen, type XVI, alpha 1 |
| 1431314_a_at | AK017936 | 5830417I10Rik | RIKEN cDNA 5830417I10 gene |
| 1433756_at | BI452674 | S100pbp | S100P binding protein |
| 1438164_x_at | BB118974 | Flot2 | flotillin 2 |
| 1448568_a_at | NM_015747 | Slc20a1 | solute carrier family 20, member 1 |
| 1450054_at | BF140063 | Add1 | adducin 1 (alpha) |
| 1452679_at | AA986082 | Tubb2b | tubulin, beta 2b |
| 1460199_a_at | BE688382 | LOC100046589 /// Pafah1b1 | platelet-activating factor acetylhydrolase, isoform 1b, beta1 subunit /// similar to platelet-activating factor acetylhydrolase 45K chain |
| 1460734_at | BG074456 | Col9a3 | collagen, type IX, alpha 3 |
| **Cluster *vi*** |  |  |  |
| 1416006_at | M34328 | Mdk | midkine |
| 1416236_a_at | BC015076 | Mpzl2 | myelin protein zero-like 2 |
| 1416237_at | BC015076 | Mpzl2 | myelin protein zero-like 2 |
| 1416532_at | NM_133901 | Trrap | transformation/transcription domain-associated protein |
| 1416686_at | BC021352 | Plod2 | procollagen lysine, 2-oxoglutarate 5-dioxygenase 2 |
| 1417385_at | AK010446 | Npepps | aminopeptidase puromycin sensitive |
| 1417654_at | BC005679 | Sdc4 | syndecan 4 |
| 1417818_at | BC014727 | Wwtr1 | WW domain containing transcription regulator 1 |
| 1417959_at | NM_026131 | Pdlim7 | PDZ and LIM domain 7 |
| 1418390_at | BB094173 | Phf21a | PHD finger protein 21A |
| 1418476_at | NM_018827 | Crlf1 | cytokine receptor-like factor 1 |
| 1419112_at | NM_008702 | Nlk | nemo like kinase |
| 1419866_s_at | AW544490 | Atxn2 | ataxin 2 |
| 1420682_at | M14537 | Chrnb1 | cholinergic receptor, nicotinic, beta polypeptide 1 (muscle) |
| 1420981_a_at | NM_010723 | Lmo4 | LIM domain only 4 |
| 1421129_a_at | NM_016745 | Atp2a3 | ATPase, Ca++ transporting, ubiquitous |
| 1422889_at | BM218630 | Pcdh18 | protocadherin 18 |
| 1422890_at | BM218630 | Pcdh18 | protocadherin 18 |
| 1423306_at | BI963682 | 2010002N04Rik | RIKEN cDNA 2010002N04 gene |
| 1423428_at | AV324603 | Ror2 | receptor tyrosine kinase-like orphan receptor 2 |
| 1423450_a_at | AV226060 | Hs3st1 | heparan sulfate (glucosamine) 3-O-sulfotransferase 1 |
| 1423694_at | BC006935 | Kctd10 | potassium channel tetramerisation domain containing 10 |
| 1424275_s_at | BC020156 | Trim41 | tripartite motif-containing 41 |
| 1424402_at | AW494299 | Rufy3 | RUN and FYVE domain containing 3 |
| 1424659_at | BG963150 | Slit2 | slit homolog 2 (Drosophila) |
| 1426778_at | BG094386 | Dag1 | dystroglycan 1 |
| 1426779_x_at | BG094386 | Dag1 | dystroglycan 1 |
| 1426858_at | BB253137 | Inhbb | inhibin beta-B |
| 1427247_at | BB238462 | D3Bwg0562e | DNA segment, Chr 3, Brigham & Women's Genetics 0562 expressed |
| 1427283_at | AK017541 | Mll1 | myeloid/lymphoid or mixed-lineage leukemia 1 |
| 1428449_at | AV297256 | Gtf3c2 | general transcription factor IIIC, polypeptide 2, beta |
| 1431055_a_at | AK010399 | Snx10 | sorting nexin 10 |
| 1434432_at | AW123157 | Rffl | ring finger and FYVE like domain containing protein |
| 1434557_at | BB794880 | Hip1 | huntingtin interacting protein 1 |
| 1437382_at | BG066107 | Acvr2a | Activin receptor IIA |
| 1437390_x_at | AV339210 | Stx1a | syntaxin 1A (brain) |
| 1438559_x_at | BB145101 | Slc44a2 | solute carrier family 44, member 2 |
| 1445689_at | AA717264 |  | Transcribed locus |
| 1448265_x_at | BC015076 | Mpzl2 | myelin protein zero-like 2 |
| 1448460_at | NM_007394 | Acvr1 | activin A receptor, type 1 |
| 1448562_at | NM_009477 | Upp1 | uridine phosphorylase 1 |
| 1448603_at | NM_009274 | Srpk2 | serine/arginine-rich protein specific kinase 2 |
| 1448870_at | AF022889 | Ltbp1 | latent transforming growth factor beta binding protein 1 |
| 1449041_a_at | NM_011639 | Trip6 | thyroid hormone receptor interactor 6 |
| 1449632_s_at | AI325255 | Fkbp10 | FK506 binding protein 10 |
| 1450140_a_at | NM_009877 | Cdkn2a | cyclin-dependent kinase inhibitor 2A |
| 1450186_s_at | NM_138658 | Gnas | GNAS (guanine nucleotide binding protein, alpha stimulating) complex locus |
| 1451224_at | BC018613 | Scamp5 | secretory carrier membrane protein 5 |
| 1452249_at | BC022643 | Prickle1 | prickle like 1 (Drosophila) |
| 1452387_a_at | AI156725 | Amotl2 | angiomotin like 2 |
| 1452632_at | BG229246 | Aak1 | AP2 associated kinase 1 |
| 1454219_at | AK013295 | Dnajc2 | DnaJ (Hsp40) homolog, subfamily C, member 2 |
| 1456131_x_at | BB131357 | Dag1 | dystroglycan 1 |
| 1456495_s_at | BG070848 | Osbpl6 | oxysterol binding protein-like 6 |
| 1456739_x_at | BB392869 | Armcx2 | armadillo repeat containing, X-linked 2 |
| 1460693_a_at | BG074456 | Col9a3 | collagen, type IX, alpha 3 |
