# Supplementary material for: Comparison of Effects of p53 Null and Gain-of-Function Mutations on Salivary Tumors in MMTV-Hras Transgenic Mice
Source: PLoS One. 2015 Feb 19;10(2):e0118029. doi: 10.1371/journal.pone.0118029 (PMC4335025; doi:10.1371/journal.pone.0118029)
Supplement: S4 Table — (DOCX) [file pone.0118029.s009.docx]

**S4 Table. Genes previously reported as regulated by p53**

| **Gene symble** | **Gene name** | **Reported regulation by p53** | **Observed regulation by p53** | **Reference** |
| --- | --- | --- | --- | --- |
| Mdm2 | Mouse double minute 2 | Induced | Induced | [[1](#_ENREF_1)] |
| Cdkn1a | Cyclin-dependent kinase inhibitor 1A (p21) | Induced | Induced | [[2](#_ENREF_2)] |
| Ccng1 | Cyclin G1 | Induced | Induced | [[3](#_ENREF_3)] |
| Igf1 | Insulin-like growth factor 1 | Induced | Induced | [[4](#_ENREF_4)] |
| Ltbp1 | Latent transforming growth factor beta binding protein 1 | Induced | Repressed | [[5](#_ENREF_5)] |
| Hs3st1 | Heparan sulfate 3-O-sulfotransferase 1 | Repressed | Repressed | [[6](#_ENREF_6)] |
| Amotl2 | Angiomotin like 2 | Induced | Repressed | [[7](#_ENREF_7)] |
| Cabc1 | Chaperone, ABC1 activity of bc1 complex homolog (S. pombe) | Induced | Induced | [[8](#_ENREF_8)] |
| Zmat3 | Zinc finger matrin type 3 | Induced | Induced | [[9](#_ENREF_9)] |

**REFERENCES:**

1. Perry ME, Piette J, Zawadzki JA, Harvey D, Levine AJ (1993) The mdm-2 gene is induced in response to UV light in a p53-dependent manner. Proc Natl Acad Sci U S A 90: 11623-11627.

2. el-Deiry WS, Tokino T, Velculescu VE, Levy DB, Parsons R, et al. (1993) WAF1, a potential mediator of p53 tumor suppression. Cell 75: 817-825.

3. Okamoto K, Beach D (1994) Cyclin G is a transcriptional target of the p53 tumor suppressor protein. Embo J 13: 4816-4822.

4. Yoon H, Liyanarachchi S, Wright FA, Davuluri R, Lockman JC, et al. (2002) Gene expression profiling of isogenic cells with different TP53 gene dosage reveals numerous genes that are affected by TP53 dosage and identifies CSPG2 as a direct target of p53. Proc Natl Acad Sci U S A 99: 15632-15637.

5. Fontemaggi G, Kela I, Amariglio N, Rechavi G, Krishnamurthy J, et al. (2002) Identification of direct p73 target genes combining DNA microarray and chromatin immunoprecipitation analyses. J Biol Chem 277: 43359-43368.

6. Daoud SS, Munson PJ, Reinhold W, Young L, Prabhu VV, et al. (2003) Impact of p53 knockout and topotecan treatment on gene expression profiles in human colon carcinoma cells: a pharmacogenomic study. Cancer Res 63: 2782-2793.

7. Liu T, Laurell C, Selivanova G, Lundeberg J, Nilsson P, et al. (2007) Hypoxia induces p53-dependent transactivation and Fas/CD95-dependent apoptosis. Cell Death Differ 14: 411-421.

8. Iiizumi M, Arakawa H, Mori T, Ando A, Nakamura Y (2002) Isolation of a novel gene, CABC1, encoding a mitochondrial protein that is highly homologous to yeast activity of bc1 complex. Cancer Res 62: 1246-1250.

9. Varmeh-Ziaie S, Okan I, Wang Y, Magnusson KP, Warthoe P, et al. (1997) Wig-1, a new p53-induced gene encoding a zinc finger protein. Oncogene 15: 2699-2704.
