# Supplementary material for: Comparison of Effects of p53 Null and Gain-of-Function Mutations on Salivary Tumors in MMTV-Hras Transgenic Mice
Source: PLoS One. 2015 Feb 19;10(2):e0118029. doi: 10.1371/journal.pone.0118029 (PMC4335025; doi:10.1371/journal.pone.0118029)
Supplement: S5 Table — (DOCX) [file pone.0118029.s010.docx]

**S5 Table. Top enriched biological functions from WT-p53 vs. p53-null comparison**

| ***Top Bio Functions*** | | |
| --- | --- | --- |
| **Diseases and Disorders** | | |
| Name | p-value | # Molecules |
| Cancer | 2.34E-06 – 3.21E-03 | 26 |
| Reproductive System Disease | 2.34E-06 – 2.57E-03 | 11 |
| Skeletal and Muscular Disorders | 5.63E-06 – 3.05E-03 | 21 |
| Respiratory Disease | 9.39E-06 – 1.85E-03 | 7 |
| Hematological Disease | 2.54E-05 – 2.72E-03 | 11 |
| **Molecular and Cellular Functions** | | |
| Name | p-value | # Molecules |
| Cell Cycle | 2.34E-06 – 3.21E-03 | 21 |
| Cellular Development | 7.42E-06 – 3.21E-03 | 17 |
| Cell Death | 2.54E-05 – 3.09E-03 | 25 |
| Gene expression | 9.04E-05 – 2.67E-03 | 20 |
| Cellular Growth and Proliferation | 9.81E-05 – 3.21E-03 | 18 |
| **Physiological System Development and Function** | | |
| Name | p-value | # Molecules |
| Tumor Morphology | 5.63E-06 – 2.57E-03 | 7 |
| Connective Tissue Development and Function | 7.42E-06 – 3.21E-03 | 17 |
| Skeletal and Muscular System Development and Function | 7.42E-06 – 2.08E-03 | 12 |
| Behavior | 4.80E-05 – 1.40E-03 | 3 |
| Reproductive System Development and Function | 4.80E-05 – 1.85E-03 | 6 |
