# Supplementary material for: Comparison of Effects of p53 Null and Gain-of-Function Mutations on Salivary Tumors in MMTV-Hras Transgenic Mice
Source: PLoS One. 2015 Feb 19;10(2):e0118029. doi: 10.1371/journal.pone.0118029 (PMC4335025; doi:10.1371/journal.pone.0118029)
Supplement: S6 Table — (DOCX) [file pone.0118029.s011.docx]

**S6 Table. Number of significant genes from different probe-level data summarization algorithms and statistical approaches**

|  | **D-Chip** | | **RMA** | | **MAS5** | |
| --- | --- | --- | --- | --- | --- | --- |
|  | WT vs KO | MT vs KO | WT vs KO | MT vs KO | WT vs KO | MT vs KO |
| t-test (5%FDR) | 21 | 0 | 10 | 0 | 6 | 0 |
| t-test (10%FDR) | 1576 | 0 | 366 | 0 | 216 | 0 |
| SAM (1%FDR) | 968 | 4 | 125 | 0 | 21 | 0 |
| SAM (5%FDR) | 4576 | 11 | 2075 | 11 | 23 | 0 |
| SAM (10%FDR) | 6892 | 170 | 4247 | 18 | 1761 | 8 |
