# Supplementary material for: Comparison of Effects of p53 Null and Gain-of-Function Mutations on Salivary Tumors in MMTV-Hras Transgenic Mice
Source: PLoS One. 2015 Feb 19;10(2):e0118029. doi: 10.1371/journal.pone.0118029 (PMC4335025; doi:10.1371/journal.pone.0118029)
Supplement: S7 Table — (DOCX) [file pone.0118029.s012.docx]

**S7 Table. Primers used in the quantitative PCR assays**

| **Gene** | **Primer** | **Sequence** | **Product size (bp)** |
| --- | --- | --- | --- |
| Cap1 | Forward | CTACCGAAGGCGGTGATTTTAAC | 114 |
|  | Reverse | ATGTATTTGCTTATCCAGCGATTTC |  |
| p53 | Forward | ATGGAGAGTATTTCACCCTCAAGATC | 101 |
|  | Reverse | CTGTAGCATGGGCATCCTTTAAC |  |
| β-actin | Forward | TCCTAGCACCATGAAGATCAAGATC | 118 |
|  | Reverse | CTGCTTGCTGATCCACATCTG |  |
