# Supplementary material for: Comparison of Effects of p53 Null and Gain-of-Function Mutations on Salivary Tumors in MMTV-Hras Transgenic Mice
Source: PLoS One. 2015 Feb 19;10(2):e0118029. doi: 10.1371/journal.pone.0118029 (PMC4335025; doi:10.1371/journal.pone.0118029)
Supplement: S1 Methods — (DOCX) [file pone.0118029.s013.docx]

**PCR primers used for mouse genotyping**

The presence of the MMTV-*ras* transgene was detected using the primers 5’-CAGGGACCAGCAAGACATC-3’ (sense), and 5’-CCCTGAACCACGCATCAAC-3’ (antisense), which generates a 723 bp PCR product in transgenic mice. Determination of the *p53* genotype (i.e., detection of *p53^+^*, *p53^-^*, and *p53^R172H^* alleles) was performed using a combination of three primers: a common exon 5 antisense primer, 5’-CCGTCATGTGCTGTGACTTCTTGTAG-3’, a *p53^+^* and *p53^R172H^*-specific intron 4 sense primer, 5’-TGTGTTGGCCATCTCTGTGAGTTCG-3’, and a *p53^-^*-specific *neo* sense primer, 5’-TCTCACCTTGCTCCTGCCGAGAAAG-3’, giving rise to products of 402 bp for the *p53^+^* allele, 519 bp for the *p53^-^* allele, and 450 bp for the *p53^R172H^* allele. The PCR products were then separated on a 2.5% agarose gel.

**DNA sequence analysis of the *p53* locus from both mouse tail and tumor tissue**

To confirm the presence of the R172H mutation in the knock-in mice, DNA spanning *p53* exon 5 was amplified from the tail DNA of a *p53^R172H/R172H^* mouse using an intron 4 sense primer, 5’-GAAAGGTCCCAGTCCTCTCTTTGC-3’ and an exon 6 anti-sense primer, 5’-CTGTCTTCCAGATACTCGGGATAC-3’. After the PCR products were separated on a 1% agarose gel, the corresponding bands were excised, purified using a GENECLEAN SPIN kit (Qbiogen Inc.), and sequenced using the same pair of primers from both directions to confirm the presence of the CGC to CAC mutation (arginine to histidine). To confirm that there were no somatic mutations in the DNA binding domain of the p53 gene in the MMTV-*ras/p53^+/+^* tumors, DNA was extracted from four salivary tumors (RAS 91, RAS 94, RAS 226, and RAS 1442, all of which were used in the microarray analysis) and the following primer pairs were used to amplify sequences spanning Exon 5-9: 1^st^ pair: sense 5’-GAAAGGTCCCAGTCCTCTCTTTGC-3’ (intron 4) and anti-sense 5’-GACTGCTGTTAAAGTAGACCCTG-3’ (intron 6); 2^nd^ pair: sense 5’-TAGCCAGCCATTCCCGGCTGC-3’ (intron 6) and anti-sense 5’-ACCTGGCAACCTGCTAATAACAC-3’ (intron 9). The amplified DNA fragments were purified and sequenced as described above, using the intron 4, intron 6 and intron 9 primers. All the resulting sequences were found to match the wild-type *p53* genomic sequence in the GeneBank database.

**Histopathological analysis**

A set of histopathological parameters were evaluated on each tumor sample in a randomized fashion and the examiner was blinded with regard to the genotypic categories. Some of the parameters were quantitated according to a 3-point grading system, including nuclear/cytoplasmic ratio, pleomorphism, and architecture. Levels of mitosis were expressed as average number of cells in mitosis over five 400X high power fields. Presence/absence of spindling morphology, apoptosis, and “giant cells” (cells with remarkably enlarged cell/nucleus size) were also evaluated for each sample. Levels of necrosis were estimated and assigned to three categories: <5%, 5-25%, and 25-50%.

**Gene expression profiling by microarray analysis**

Tumors used for microarray analysis were selected from the archived frozen collection according to the following criteria: (1) size: relatively small tumors (~400-800 mg) were used for microarray analysis to minimize tumor heterogeneity; (2) growth rates: tumors whose growth rates were consistent with most of the other tumors in the same genotypic group; (3) histology: tumors with the typical morphological features of adenocarcinoma and lacking extensive necrosis. For RNA extraction, 20-40 10μm frozen sections from each candidate tumor were collected. After every 10 sections, a 5μm section was collected, stained with hematoxylin and eosin, and examined microscopically. Only samples showing greater than 85-90% of homogeneous tumor mass were selected for the subsequent microarray study. RNA was extracted using TRIZOL reagent (Invitrogen Life Technologies, Carlsbad, CA), according to the manufacture’s protocols. Total RNA isolated from the tumor sections was subjected to a cleanup step using the RNeasy Mini Kit (Qiagen Sciences Inc., Germantown, MD). The purity of the RNA preparation was judged as adequate if the A_260_/A_280_ ratio was ≥2.0 and the A_260_/A_270_ ratio was ≥1.3. RNA integrity was examined by running 1μl of the RNA sample on an RNA 6000 Nano LabChip in the 2100 Bioanalyzer (Agilent, Palo Alto, CA). A ribosomal RNA 28S/18S ratio of ≥1.5, with the rRNA contribution being 30% or more, was considered satisfactory. Microarray analysis was performed using the Affymetrix GeneChip platform and the Mouse 430A 2.0 array, which has 22,690 probe sets representing over 14,000 unique mouse genes. The Affymetrix standard protocol has been described extensively [50; 51]. Briefly, at least 5μg total RNA was converted to cDNA using the SuperScript Choice System (Life Technologies, Rockville, MD), in which first strand cDNA synthesis was primed with a 24-mer oligo(dT) with a T7 RNA polymerase promoter site attached to the 3’ end. cDNA was extracted with phenol/chloroform and precipitated in ethanol. Using 2μg cDNA, biotin-labeled cRNA was synthesized using the Enzo BioArray High Yield RNA Transcript Labeling Kit (Enzo Diagnostic Inc., Farmingdale, NY) with Bio-11-CTP and Bio-16-UTP. The cRNA was subjected to a cleanup step using the RNeasy Mini Kit (Qiagen Sciences Inc., Germantown, MD), and was then fragmented. 20μg of the fragmented cRNA from each sample was hybridized to the Mouse 430A 2.0 chip for 18 to 20 hours. The chip was then washed and stained with streptavidin phycoerythrin (SAPE; Molecular Probes, Eugene, OR) added with an antistreptavidin biotinylated antibody (Vector Laboratories, Burlingame, CA). Each chip was scanned at a 6-μm resolution by the Agilent G2500A Technologies Gene Array scanner (Agilent Technologies, Palo Alto, CA). After scanning, the raw intensities of each probe were stored in electronic files (in .DAT and .CEL formats) by the Microarray Suite 5.0 software (Affymetrix, Santa Clara, CA).

**Microarray data analysis**

Multiple levels of data analysis were performed in the BRB-ArrayTools software, version 3.5.0 (Richard Simon & Amy Peng Lam, Biometric Research Branch, Division of Cancer Treatment and Diagnosis, NCI), which is an integrated software package for visualization and analysis of DNA microarray gene expression data. The probe-level microarray data (in .CEL files) were collated in BRB-ArrayTools using the RMA (Robust Multi-chip Average) method, which involves a background correction for the PM (Perfect Match) data, a quantile normalization [52], and summarization of the expression levels using the Tukey’s median polish algorithm on an additive linear model [53]. The estimated expression levels from the above processes are in the base-2 logarithm format and these values were used in the subsequent statistical analysis. Alternatively, they were also transformed back to the original scale by using the 2^(log value) function for visualization purpose and all the histograms of expression level were derived based on the original scale. During data collation, no filtering option was chosen, so expression levels were summarized for all the 22,690 probe sets. In all the analysis thereafter, a subset of 27 quality control probe sets were excluded (Table 2), resulting in 22,663 probe sets used.

The integrated clustering function in BRB-ArrayTools was used to cluster genes or samples or both, and generate heat maps of the results. Hierarchical clustering was carried out in either an unsupervised fashion for which all the 22,663 probe sets were used, or a supervised fashion for which user-defined gene lists were used. All the cluster analyses were performed by choosing the Pearson correlation coefficient (called “One minus correlation” in BRB-ArrayTools) and average linkage options.

To identify genes differentially expressed between different groups of tumor samples, with defined statistical significance, the Class Comparison Between Groups of Arrays tool of the BRB-ArrayTools software was used. The software provides options including the random variance version of the *t*-test, with sub-options of performing either univariate permutation tests or multivariate permutation tests, and the Statistical Analysis of Microarrays (SAM) method. The SAM algorithm [54] is a permutation-based method of controlling the False Discovery Rate (FDR) with a less stringent statistical basis than the similar multivariate permutation test. For all the class comparison analysis in this study, SAM was used.

**Functional analysis of microarray data**

To explore the biological meanings of the identified lists of significant genes from different class comparison analysis, two software packages designed for gene function analysis were used.EASE (http://david.abcc.ncifcrf.gov/) is a program designed to discover Gene Ontology (GO) categories that are over-represented in the chosen gene list compared to what is represented in the microarray or species genome as a whole. Such over-represented categories represent biological “themes” of a given list. Each identified GO category is assigned an EASE Score, which is the upper bound of the distribution of Jackknife Fisher exact probabilities given the List Hits (number of genes in the gene list that belong to the Gene Category), List Total (number of genes in the gene list), Population Hits (number of genes in the total group of genes assayed that belong to the specific Gene Category) and Population Total (number of genes in the total group of genes assayed that belong to any Gene Category within the System).

Ingenuity Pathways Analysis (IPA) (Ingenuity Systems, http://www.ingenuity.com) is a software application based on a knowledge repository that houses the biological and chemical relationships extracted from the scientific literature and enables users to identify relevant gene networks from the microarray expression data. It also works as a database for quickly identifying functional annotations of a single gene or a list of genes. The significance value associated with the functions and pathways identified by the analysis is a measure for how likely it is that genes in the differential expression list participate in that function. The significance is expressed as a p-value, which is calculated using the right-tailed Fisher's Exact Test. In this method, the p-value is calculated by comparing the number of genes from the microarray analysis (i.e. Functional Analysis Genes) that participate in a given function or pathway, relative to the total number of occurrences of these genes in all functional/pathway annotations stored in the Ingenuity Pathways Knowledge Base.

**Validation of microarray data by quantitative PCR**

The first batch of total RNA used for microarray experiments and a second batch of total RNA from the same set of tumor samples, isolated the same way as the first batch but at a later time point, were used for quantitative PCR analysis. We chose the two-step RT-PCR approach, in which total RNA was first subjected to cDNA synthesis and the cDNA was used for real-time PCR amplification in two separate steps. 3μg of total RNA from each tumor sample was loaded in each RT reaction, using the SuperScript III First-strand Synthesis system (Invitrogen Inc., Carlsbad, CA) according to the manufacture’s protocol, and oligo(dT) was selected to prime the RT reactions. cDNA samples from the RT reactions were diluted 50X and stored at -20°C as stock cDNA solutions for the following real-time PCR reactions. Undiluted cDNA from three tumor samples representing the three genotypic groups was used in serial dilutions to generate standard curves in the quantitative assay. Primers for the real-time PCR assays were designed using the Primer Express Version 3.0 software (Applied Biosystems) using the standard melting temperature (60°C) and at least one of the two primers for each gene was selected to span an Exon-intron boundary, to avoid amplifying genomic DNA contaminants. The real-time PCR assay was performed on the Applied Biosystems 7500 Real-time PCR System (Applied Biosystems). In a 15μl reaction, 5μl of the sample cDNA was mixed with the primers and iTaq SYBR Green Supermix (Bio-rad), in which SYBR Green works as the reporter dye and ROX as the passive reference. The final concentration of the primers used for each gene was determined by preliminary assays as to give the highest amplification efficiency but not significant formation of primer-dimers. The Relative Standard Curves method was used as the study design for the quantitative PCR assay (Real-Time PCR Systems Chemistry Guide, Applied Biosystems). Beta-actin was selected as the internal control. Five 2X serial dilutions were applied (1:10, 1:20, 1:40, 1:80, and 1:160) to generate a standard curve for each primer pair on each plate. The raw data were analyzed in the Sequence Detection Software Version 1.3.1 (Applied Biosystems) according to the manufacture’s protocol. Sequences of the primers used in the quantitative PCR assays are listed in Suppl. Table 7.
